# Nanotechnology-Based Strategies to Evaluate and Counteract Cancer Metastasis and Neoangiogenesis

**DOI:** 10.1002/adhm.202002163

**Published:** 2021-03-24

**Authors:** Özlem Şen, Melis Emanet, Gianni Ciofani

**Affiliations:** Istituto Italiano di Tecnologia Smart Bio-Interfaces Viale Rinaldo Piaggio 34, Pontedera, Pisa 56025, Italy; Sabanci University Nanotechnology Research and Application Center (SUNUM) Sabanci University Universite Caddesi 27-1, Tuzla, Istanbul 34956, Turkey; Istituto Italiano di Tecnologia Smart Bio-Interfaces Viale Rinaldo Piaggio 34, Pontedera, Pisa 56025, Italy

**Keywords:** angiogenesis, cancer, invasion, metastasis, nanomaterials

## Abstract

Cancer metastasis is the major cause of cancer-related morbidity and mortality. It represents one of the greatest challenges in cancer therapy, both because of the ability of metastatic cells to spread into different organs, and because of the consequent heterogeneity that characterizes primary and metastatic tumors. Nanomaterials can potentially be used as targeting or detection agents owing to unique chemical and physical features that allow tailored and tunable theranostic functions. This review highlights nanomaterial-based approaches in the detection and treatment of cancer metastasis, with a special focus on the evaluation of nanostructure effects on cell migration, invasion, and angiogenesis in the tumor microenvironment.

## 1 Introduction

Cancer is one of the foremost health problems worldwide, and the vast majority of cancer deaths (≈90%) are caused by metastatic diseases rather than primary tumors.^[1]^ The spread of cancer cells from a primary tumor to the surrounding tissue or distant sites to seed secondary tumors, called metastasis, is the greatest cause of failure of cancer therapy.^[2]^ The formation of metastatic niches begins with the loss of malignant cell adhesion, conferring the ability to enter the bloodstream. As these cells search for a new home tissue, they spread through the circulatory system and adhere to the vascular walls. They pass though the endothelial barrier, extravasate at a distant site, and finally infiltrate a new tissue. Several biochemical and biophysical processes participate in this cascade, such as changes in cell polarity, the cytoskeleton, and the expression of membrane proteins.^[3]^ Metastasis has already occurred in a number of patients even before they are diagnosed with primary cancer, and shows several modalities depending on the cancer type. For example, it is difficult to detect metastasis in breast cancer, as it can remain latent for years, while lung cancer metastasis has often formed in multiple organs at the time of initial diagnosis. In addition, conventional treatment strategies such as surgical removal, chemotherapy, and radiotherapy can be applied successfully to only a small number of patients diagnosed with metastatic cancer.^[4]^

Nanotechnology represents a wide field with exponential growth and enormous potential in cancer treatment, already used in the clinic. For example, the U.S. Food and Drug Administration (FDA)-approved liposomal doxorubicin (Doxil) is widely used in ovarian cancer and in Kaposi’s sarcoma with low cardiotoxicity due to the encapsulation of the drug,^[5]^ and Abraxane, a paclitaxel-containing protein nanoparticle, is used to treat metastatic breast cancer.^[6]^ Altogether, it is believed that nanotherapeutics will introduce new strategies to treat or prevent metastasis, including earlier detection and superior targeting to metastatic sites.^[7]^

This review highlights nanomaterial-based strategies, and specifically those associated with inorganic (gold, magnetic, quantum dots, silica, and carbon-based), polymer-based (natural and synthetic), and lipid-based nanomaterials (liposomes, solid lipid nanoparticles, nanostructured lipid carriers), to counteract cancer metastasis and angiogenesis. First, a brief summary of cancer metastasis, the origin of metastatic cancer cells, and cancer-related angiogenesis is presented, followed by an overview of the main assays used to evaluate cancer metastasis and angiogenesis; the second part of this Review is thereafter dedicated to a discussion of nanomaterial-mediated treatments. Finally, the conclusions highlight the current status and future prospects of the efforts to counteract the metastatic spread of malignant tumors.

## 2 Cancer Metastasis and Angiogenesis

Cancer is a major health burden worldwide; it is estimated that 1 806 590 new invasive cancer cases and 606 520 cancer-related deaths will occur just in the United States in 2020.^[8]^ Cancer can be defined as a group of diseases characterized by the multiplication of abnormal cells, which have the ability to infiltrate normal body tissue and escape the natural mechanism of cell death. It has a number of key features derived from genetic instability, including the avoidance of apoptosis and growth suppression, unlimited replicative potential, the induction of angiogenesis, and the activation of invasion and metastasis.^[9]^ Repeated exposure to carcinogens, such as ultraviolet light, tobacco smoke, insistent tissue damage, and some viral infections, cause genetic and epigenetic changes leading to cancer initiation, progression, and metastasis.^[10]^

### 2.1 Metastasis

**Figure 1:**
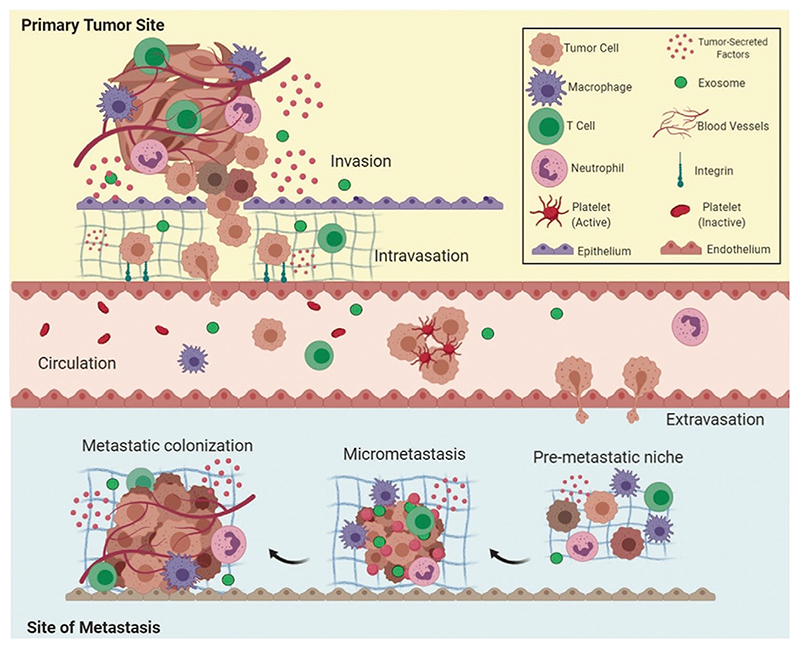
Overview of the metastatic cascade. The five key steps of metastasis are depicted: invasion, intravasation, circulation, extravasation, and colonization. Reproduced under the terms of the CCA 4.0 International Licence.^[12]^ Copyright 2020, Springer Nature.

Cancer metastasis is the spread of cancer cells, which from the primary tumor circulate, settle, and grow in a new area. Metastasis has already occurred for most patients before being diagnosed with cancer, and is the major cause of morbidity and mortality, being responsible for ≈90% of cancer deaths.^[11]^ Metastases develop when cancer cells leave their primary sites, travel through the bloodstream, withstand the pressure in blood vessels, acclimate to the new cellular environment in a secondary area, and escape from immune cells. Figure 1 shows the five key steps of metastasis: invasion, intravasation, circulation, extravasation, and colonization.^[12]^

The initial trigger for the dissemination and invasion of cells is chromosomal instability: continuous defects in chromosomal segregation lead to the initiation of the process.^[13]^ Then, the process of movement through the basement membrane into a blood or lymphatic vessel, intravasation, triggers the development of a distant metastasis. Other cells in the tumor microenvironment, signal molecules, and proteases affect cancer cell intravasation,^[14]^ that can be active or passive depending on the tumor type, microenvironment, and vascularization.^[15]^ Cells can circulate as single cells or as cell clusters; circulatory travel is harsh, and it generally determines the fate of most in-travasating cancer cells: survival or death. Most of the cells die in the bloodstream due to physical/oxidative stress, anoikis (a form of programmed cell death), and a lack of cytokines and growth factors. If they survive, they actively extravasate into surrounding sites or become trapped in capillaries:^[16,17]^ this results into microvascular rupture or extravasation.^[12]^ Generally, extravasated tumor cells undergo one of three alternative routes: cell death, dormancy (survival without a significant proliferation), or colonization.^[18]^ The growth of the metastatic colony is the last and most lethal stage in the malignant progression of a tumor, while the precolonization stage of metastasis involves a series of events occurring over a time scale ranging from minutes to hours.^[19]^

Although the key steps of metastatic cascade are well-known, the process through which the metastatic cells arise from the nonmetastatic cells populations of the primary tumor is largely unclear. Epithelial-mesenchymal transition (EMT) is a highly conserved biological process for morphogenesis during embryonic development. It relies on the transition between the epithelium and mesenchyme, and plays a role in gastrulation and in heart and neural crest formation.^[20]^ However, this significant and fundamental developmental process in multicellular organisms has a more sinister role in tumorigenesis. It is assumed that metastatic cells originate from epithelial stem cells or differentiated epithelial cells through a gradual accumulation of gene mutations, that eventually transform epithelial cells into tumor cells with mesenchymal properties.^[21]^

At the beginning and progression of carcinoma, normal epithelial cells can proliferate to form an adenoma; however, the genetic and epigenetic changes leading to an in situ carcinoma still outline an intact basement membrane. Further changes may enhance the spread of carcinoma cells via E MT, resulting in fragmentation of the basement membrane. This leads to intravasa-tion of the cells into blood or lymph capillaries through passive transport to distant organs. Furthermore, solitary cells may either remain solitary or form a new carcinoma via mesenchymal-epithelial transition.^[22]^

The second possibility of origin of cancer metastasis is mediated by cancer stem cells (CSCs). Evidence for a role of CSCs was proposed for the first time in 1994 by Lapidot et al.^[23]^ Many attempts have thereafter shown that metastasis is driven by CSCs.^[24–26]^ Since stem cells are known for their ability to proliferate and migrate during tissue morphogenesis and differentiation, it can be assumed that genetic damage to stem cells could lead to metastatic cancers in various tissues.^[21]^ Cancer cells can differentiate into CSCs as a result of cellular stress or therapy; thereafter, CSCs can differentiate into all other cancer cell types, such as endothelial cells and cancer-associated fibroblasts.^[27]^ It was also suggested that CSCs can transform to migrating stem cells through EMT and then form metastases.^[28]^ Although many studies have been performed on CSCs, whether they are present in all human tumors is still unclear, and the characterization of these cells in most tumor types remains elusive.

**Figure 2:**
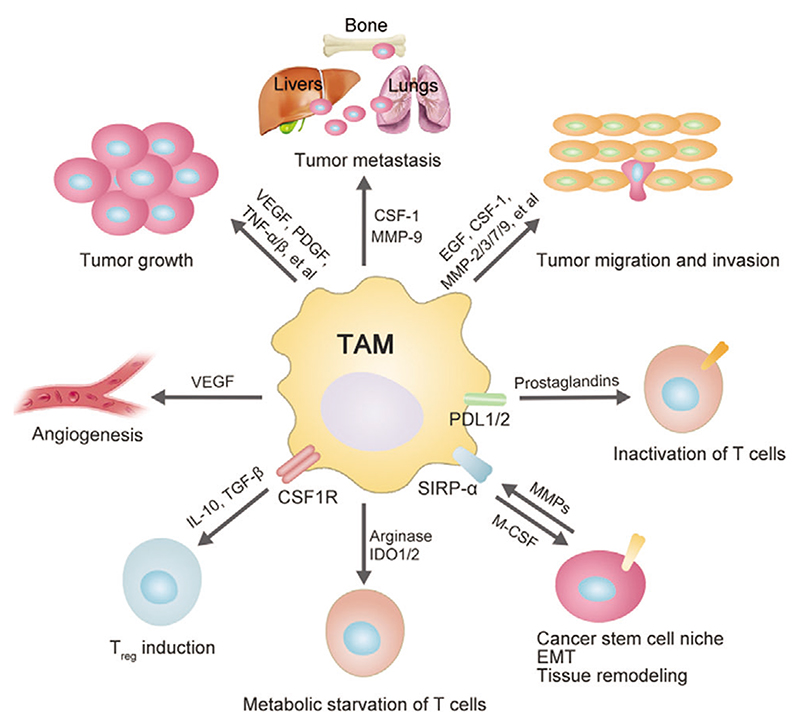
Major roles of TAMs in tumor progression. Reproduced under the terms of the CCA 4.0 International Licence.^[32]^ Copyright 2019, Springer Nature.

Another hypothesis on the origin of cancer metastasis is related to macrophages, which are a type of versatile immuno-cytes that can clear harmful and foreign substances, including cellular debris and tumor cells.^[29]^ In the microenvironment of solid tumors, one of the most abundant immune cell typology is represented by macrophages.^[30]^ Tumor-associated macrophages (TAMs) promote metastasis by altering the composition of the extracellular matrix (ECM) via the release of several chemokines, growth factors, and inflammatory factors. TAMs can also participate in several processes in metastasis, such as EMT, intrava-sation, circulation through the bloodstream, extravasation, and survival and growth at the metastatic site.^[30,31]^ Figure 2 shows the major roles of TAMs in tumorigenesis: as depicted, the secretion of several cytokines, inflammatory substances, proteolytic enzymes, and growth factors by TAMs promotes tumor initiation, growth, development, and metastasis.^[32]^

Myeloid cells are composed of several mononuclear and polymorphonuclear phagocytes and by their precursors, such as monocytes/macrophages, granulocytes, dendritic cells, and myeloid-derived suppressor cells. They play critical roles in tumor initiation, development, and metastasis.^[33]^ The myeloid hypothesis of metastasis can be thought of as an alternative or complementary hypothesis to the tumor-associated macrophage hypothesis. It suggests that metastatic cancer cells directly originate from myeloid cells or from hybrid cells formed by the fusion of nonmetastatic stem cells and macrophages.^[21]^ It has been reported that myeloid cells promote delamination (the process by which cancer cells detach from the primary tumor mass), invasion, intravasation, extravasation, and colony formation at the metastatic site.^[34]^

### 2.2 Angiogenesis

Angiogenesis generally refers to the process of vessel growth, but most strictly refers to new vessels that stem from pre-existing vessels.^[35]^ It comprises several crucial steps, including endothelial cell proliferation, stimulation of endothelial cells by endogenous growth factors, such as vascular endothelial growth factor (VEGF), cell migration, and capillary tube formation.^[36]^ It plays an important role in embryogenesis (it is called vasculogenesis when it occurs in early embryonic development) and to a limited extent in adults.^[37]^ Although there is little turnover of endothelial cells in adult vascularization, it occurs during the menstrual cycle, ischemic tissue restoration, and wound repair.^[38]^

Cancer-related angiogenesis was first proposed in 1968, and it was emphasized that a diffusible substance from the tumor area stimulates angiogenesis.^[39]^ Angiogenesis is required for proper nutrition and removal of metabolic wastes during tumor progression;^[40]^ once the tumor starts to grow and reaches a few millimeters in diameter, an “angiogenic switch” is triggered by hypoxia and nutrient deprivation for tumor development. Then, tumor cells release cytokines and growth factors for the activation of normal/quiescent cells placed around the tumor environment, and an angiogenesis cascade is initiated.^[41]^ The angiogenic switch can be thought of as a different step from tumor progression; it occurs at different phases in tumor growth related to the tumor type and microenvironment and comprises several steps, including perivascular detachment and vessel dilation, initiation of angiogenic sprouting, new capillary formation and development, and recruitment of perivascular cells. Tumor growth is supported by new blood vessels that specifically feed necrotic and hypoxic tumor areas to supply nutrients and oxygen.^[42]^

Intratumor angiogenic development has heterogeneous formation depending on different tumor tissues and stages; tumor neovascularization is composed by a chaotic mixture of abnormal, hierarchically disorganized cells that differ from those of normal vascularization in terms of structural and functional aspects.^[43]^ Tumor vascularization is stimulated by growth factors and cytokines, including vascular permeability factor and vascular endothelial growth factor (VEGF-A), which are secreted by primary tumor cells. Following angiogenesis stimulatory agent secretion from tumor cells, degradation of vascular basement membrane around vessels occurs, thus hindering undesired vessel grow. This degradation is based on the action of proteases that attack collagen IV and laminin β1 proteins in the membrane structure. Finally, the vascular extension is allowed by the proliferation of vascular endothelial cells. The irregular cell proliferation and maturation in neovascularization, together with variations in thickness and density of extracellular matrix, generates disproportional vessel permeability named as enhanced permeability and retention (EPR) effect. Since the discovery of EPR effect, many efforts have been made to target different nanoparticles into the tumors area by exploiting this phenomenon;^[44]^ due to the uncontrolled flow of compounds through the leaky vessels, nanoparticles in fact preferentially accumulate into the tumor tissues with respect to the other tissues. Several techniques aim at enhancing the EPR effect, including angiotensin (AT)-induced hypertension, modulation of tumor vasculature through vascular disrupting agents, angiogenesis inhibition, or even photodynamic therapy (PDT).^[45–48]^

Recently, inorganic nanoparticles have been reported as tumor vessel disrupting agents able to induce vascular leakiness; this phenomenon is named NanoEL, and is attributed to the disruption of cell–cell connection induced by the degradation of interaction proteins that leads to creation of gaps among cells,^[49]^ providing paracellular passage of drug molecules across the vascular wall. Currently, several inorganic nanoparticles have been reported as alternative structures to induce NanoEL. For instance, spherical titania nanoparticles (Ti_2_NPs) have been found to disrupt the hemophilic interaction between vascular endothelial (VE)-cadherin proteins in endothelial cell structure, leading to cell retraction and development of micrometer-sized gaps between cells.^[50]^ In another study, the NanoEL effect was observed following treatment with ad hoc functionalized gold nanoparticles (AuNPs); interestingly, negatively-charged AuNPs could elicit greater NanoEL effect response because of repulsive interactions with the glycocalyx of the cell surface.^[51,52]^

### 2.3 Drugs Exploited to Counteracts Metastasis and Angiogenesis

A considerable number of drugs have proven to be efficient against cancer angiogenesis and metastasis. Some examples of antiangiogenic and antimetastatic drugs include axitinib (kidney cancer),^[53]^ cabozantinib (thyroid cancer and kidney cancer),^[54]^ everolimus (kidney cancer, advanced breast cancer, pancreatic neuroendocrine tumors-PNETs-),^[55]^ lenalidomide (multiple myeloma, lymphoma),^[56]^ and ramucirumab (advanced stomach cancer).^[57]^ In detail, bevacizumab (avastin), a monoclonal antibody that targets VEGF-A receptors and inhibits their functions, was approved in the USA and in EU as an antiangiogenic treatment for metastatic breast, lung, and colon cancer.^[58]^ Suni-tinib (Sutent) is another inhibitor that has an affinity for multiple targets, including VEGF, platelet-derived growth factor receptor (PDGFR), and tyrosine kinase 3 (FLT-3), and it has been approved for the treatment of advanced renal cancer.^[59]^ Sorafenib (Nexavar) is another multitargeted inhibitor specific for VEGF, PDGFR, and rapidly accelerated fibrosarcoma kinase, that has been approved for hepatocellular carcinoma and for advanced renal cancer.^[60]^

## 3 Common Assays for the Evaluation of Cancer Metastasis and Angiogenesis Progression

**Figure 3:**
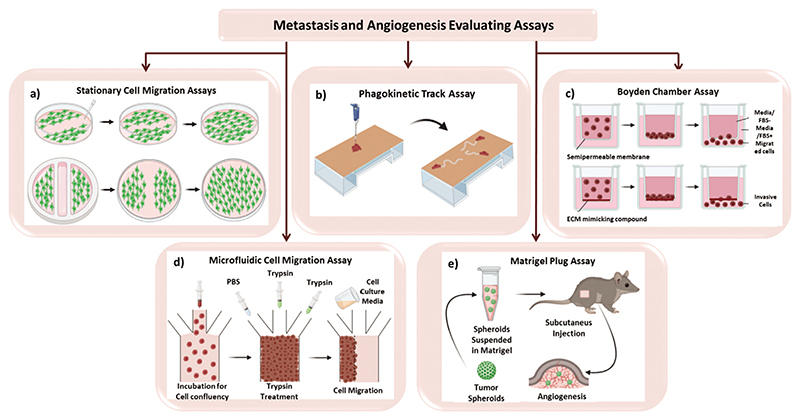
Schematic illustration showing the main assays exploited for the evaluation of metastasis and angiogenesis. a) Scratch/wound healing-based cell migration assay, b) phagokinetic track assay, c) transwell Boyden chamber assay, d) microfluidic cell migration assay, e) matrigel plug xenograft assay.

In this section, we report a brief overview of the main assays exploited for assessing cancer metastasis and neoangiogenesis (Figure 3), the most relevant of which are summarized in Table 1 and described in details in the next paragraphs.

### 3.1 Metastasis Evaluation

Tumor metastasis is a multistep process that includes local tumor cell detachment, intravasation, dissociation into the circulating blood vessels or lymphatic system, and extravasation into distal organs.^[61,62]^ Main cancer treatments are generally based on cancer cell cytotoxicity induced by targeted drugs developed to clear or reduce the size of solid tumors,^[63]^ while the underestimation of tumor invasion/metastasis phenomena and the misadministration of antimetastatic drugs dramatically decrease the success rate in cancer fighting.^[64]^

#### 3.1.1 In Vivo Assays

Metastasis detection by using in vivo assays commonly provides rough information about the final location of the cells, with very little information about intermediate steps of migration.^[65]^ New-generation imaging is the only technique used to detect cancer cells migrating to the rest of the solid tumor. Multiphoton imaging techniques utilize long wavelengths of light pulsed from lasers to excite molecules in a limited focal plane through the simultaneous absorption of two or three photons, and have a very important role in metastasis detection.^[66]^ The most important advantage of this method over conventional single-photon methods is that the exploitation of a long wavelength of light provides deeper tissue penetration, resulting into more detailed evaluation through the deepness of tissue. Although multiphoton microscopy for intravital imaging offers several advantages, the rather high cost of the necessary equipment and its still limited tissue penetration push research toward alternative imaging technique,^[67]^ such as the real-time whole-body fluorescent imaging. Recently, intravital optical frequency domain imaging (OFDI) has been used to monitor metastasis,^[68]^ while traditionally clinic approaches are represented by magnetic resonance imaging (MRI), computed tomography (CT), and positron emission tomography (PET), that are also commonly used to assess the presence of metastatic cancer cells.^[69,70]^

#### 3.1.2 In Vitro Assays

In the in vitro evaluation of metastasis, the investigation of cell adhesion capacity and of the ECM component profiles play crucial roles in revealing alterations in cell migration ability, based on parameters such as cancer type, tissue, and cellular origin. These changes contribute to tumor cell detachment from solid tumors, spread in the blood or lymphoid stream, and survival at the arrival point.^[71,72]^ Therefore, cell adhesion capacity and ECM component investigations are privileged in vitro studies for evaluating the metastatic behavior of cancer cells. On the other hand, the migrational features of cancer cells are classified as random (kinesis), growth factor or chemokines derived (chemokinesis), adhesive substrate derived (haptokinesis), or directional (taxis);^[73]^ in this regard, the difference in migrational behaviors of different cancer cells against kinesis and taxis is quite unclear, as well as their response when exposed to multiple signals.^[74]^

The 2D scratch/wound healing-based metastasis assay involves confluent cells cultured as a monolayer, and evaluates migration through the cell-free area of the plate. The ratio between wound surface area and time provides information about cell migration capacity at morphological level.^[75]^ Based on the wound (cell-free) area generation method, the assay is differently exploited: a wound area is linearly created at the center of plates by making a simple scratch with a pipette tip and an automated 384-well scratch device to prevent cell attachment or growth as shown in Figure 3a.^[76–78]^ As a cheap and easy technique, scratching the wound area provides a rough evaluation of cell migration kinetics due to the physical injury, but the ECM located at the scratch area can generate inconsistent results from the real migration profile of the in vivo cells.^[79]^ This condition can be partially overcome with the help of new generation technologies, like the Electric Cell Impedance Sensing Assay (ECIS, Applied BioPhysics), which prevents cell attachment or growth in the wound area owing to the presence of electrodes.^[80]^ On the other side, in the gap closure assay, cells are cultured in specialized wells, including an insert that is exploited to produce a gap (cell-free area) in the plate by preventing cell attachment and growth (Figure 3a, bottom). ^[79]^ An advantage of this method is represented by the fact that it gives a migration profile highly consistent with in vivo evaluations, by providing a wound area without causing any damage to the cells.^[81]^ The migration of the cells can be quantified by standard light microscopy or by using quantitative software such as MetaMorph or IncuCyte.^[82]^

In the phagokinetic track assays, the metastatic tendency of cells is evaluated basing on their migration on colloidal gold-coated cover slips without using any barrier (Figure 3b).^[83]^ The cells internalize the colloidal golds during their migration, and the loss of colloidal golds on the cover slip reveals a track that indicates the migration performance of cells. One of the disadvantage of this approach is the presence of colloidal gold, that could affect the migration capacity of cells.^[83]^

The Boyden chamber assay was elaborated by Boyden in 1962 and can be separately utilized for analyzing the migration, invasion, and transendothelial migration of cells by tailoring the filter surface. The assay consists of two nestled chambers oriented to provide contact between two environments including different biochemical compounds (Figure 3c). The upper chamber has a filter at the bottom, and is placed in contact to a chamber provided with medium containing chemotactic agents.^[84]^ The migration is evaluated by seeding the cells on the filter in the upper chamber, and their migration to the bottom chamber is monitored by light microscopy.^[85]^ In invasion assays, cells are seeded on coated filters with ECM-mimicking compounds, usually fibronectin, collagen, or Matrigel; thereafter, cell invasion through the ECM mimicking compound and their passage through the filter is evaluated;^[86]^ in a similar approach, transendothelial migration can be evaluated by considering cell migration through an endothelial cell culture.^[87]^ The migration ability of the cells in the direction of chemotactic agents through the filter in the presence or absence of fetal bovine serum as a chemotactic agent can also be evaluated; the possible technical difficulty of this method is the low stability of the transmembrane gradient in case of utilization of the coated filter in prolonged process duration.^[88]^ This approach can also be used in combination with standard in vivo selection methods that provide isolation of metastatic cells with enhanced metastatic propensity toward organs that are poorly colonized with current animal models.^[89]^

Microfluidic systems are exploited with the purpose of performing more realistic and adaptable in vitro migration assays than those obtained with 2D models and transfer chamber assays. Additionally, microfluidic systems provide the opportunity to test the effects of multiple parameters; moreover, the migration ability of the cells can be evaluated in a fluidic flow that provides continuous mechanical stimulation with a controlled biochemical environment. In these assays, the migrational behavior of cells can be evaluated in conditions mimicking the blood circulation or the lymphatic stream. In this regard, microfluidic systems have been combined with microfilter systems or hydrogel scaffolds to simulate ECM filtration.^[90]^ A further advantage is provided by the strong flexibility in controlling the environmental factors of cells, including the opportunity to tune biochemical and biomechanical conditions.

Basically, microfluidic systems involve a cell culture chamber bridged to another chamber with an internal channel loaded with medium including a chemotactic agent.^[91]^ In the plug and play microfluidic-based assays, the U-well is allowed to be inserted directly into the metastasis chip housing the complete cell culture in flow conditions.^[62]^ The cells in the first chamber are separated from the channel with a microfilter in the U-well, which differs from other methods that provide a barrier effect against cell migration in the direction of a chemotactic agent across the filter. This system also reduces the experimental setup time and avoids initial culture failures during the cell seeding process compared to other existing microfluidic metastasis chips. In addition, the cells that migrate through the channel can be quantified by staining with fluorescent dyes loaded into the second chamber, which provides a further advantage in evaluating cell migration ability without requiring a further cell staining process. Moreover, the high sensitivity of this method provides an opportunity to analyze a small volume of samples and a low number of cells; this is particularly useful in evaluating rare primary cell populations obtained by biopsy.^[62]^

In another type of microfluidic-based assays, named the on-chip cell migration assay, the fluidic channel is partially separated into several small channels to allow different fluidic solution to flow at the same time (Figure 3d).^[92]^ As an alternative wound generating system, confluent cultured cells are exposed to different fluids flowing inside small channels; since trypsin/EDTA solution flows from some of the channels, the cells are detached and leave a cell-free area. This method provides a wound edge in the border of live cells, preventing any damage to the cells located at the wound edges; cell migration through the cell-free area is monitored to follow cell metastasis ability, and cell responses to several exterior factors can be observed.^[92]^

### 3.2 Angiogenesis Evaluation

The angiogenic process basically consists of two well-synchronized pathways: splitting and sprouting. In the case of excessive stress in the microvascular system, intraluminal splitting into two vessels occurs, whereas tissue hypoxia more often leads to the sprouting of cells that cause a new linear capillary to develop from a preexisting one.^[93]^ Angiogenesis fundamentally starts from “tip cells” that exist inside capillaries, proliferate, and migrate to the end points of vessels both in splitting and sprouting phases of neovascularization.^[94]^ Then, the mural cells (pericytes and vascular smooth muscle cells) mediate for the development of new vessel walls by surrounding vascularized endothelial cells.^[95]^ All these processes are oriented by pro- and antiapoptotic factors that work in a marvelous synchronization based on the oxygen and nutrition requirements in tissues.^[96,97]^

#### 3.2.1 In Vivo Assays

The Matrigel plug assay, developed by Passaniti et al. is a relatively quick and easy method to evaluate both angiogenic and antian-giogenic compounds.^[98]^ In this assay, angiogenic generation including cell proliferation and interaction is monitored on specifically developed Matrigels extracted from an Engelbreth–Holm– Swarm mice tumor extracellular matrix without proangiogenic rich ingredients.^[99]^ In in vivo experiments, Matrigel is subcutaneously injected into C57/BL mice in liquid form at 4 °C, and solid subcutaneous Matrigel is obtained thanks to its rapid solidification at 37 °C. The new vessels become apparent in the Ma-trigel after only 2 or 3 days; however, mature vessel generation reached a maximum level at 3 weeks after Matrigel implantation. Owing to the Matrigel being initially avascular, any vessel formation indicates neovascularization. Following a one- or 3-week incubation process, the Matrigel is removed from the mice, and vessel formation is visualized by immunostaining.^[100]^ Although Matrigel provides a natural microenvironment for the angiogenesis in mice, it is a quite expensive method and is affected by conditions depending on the animal and on the site of implantation.

The sponge/Matrigel assay is a modified form of the Matrigel assay that includes a scaffold (sponge) fragment buried in the Matrigel to improve the visualization of the angiogenic response.^[101]^ To visualize the new vessels, fluorochrome-labeled high molecular weight dextran (fluorescein-5-isothiocyanate-dextran-FITC-dextran) is applied to the sponge/Matrigel. Then, formalin-fixed Matrigel plugs are monitored by phase-contrast microscopy, and perfused new vessels can be identified by UV illumination.^[102,103]^ The sponge/Matrigel assay was developed as a high magnification technique for improved visualization; however, it is more time consuming and expensive than the simple Matrigel assay.

The alginate microbead release assay is similar to the Matrigel plug technique as well; in this assay, proangiogenic factors and cancer cells are encapsulated within alginate beads and subcutaneously injected as shown in Figure 3e.^[104]^ With the help of alginate encapsulation, cancer cells do not come in contact with the surrounding tissue, and proangiogenic factor release occurs in a controllable way. Moreover, the alginate beads can protect their stability under in vivo conditions for up to 1 month, which is an adequate duration for new vessel generation.^[105]^ The angiogenic generations in alginate microbeads are quantified by measuring total hemoglobin content; however, hemoglobin-based quantification is questioned due to the relevance of hemoglobin in alginate microbeads, which change depending on the implantation site. Therefore, FITC-dextran is used to quantify vascularization by monitoring vessel formation in alginate microbeads.^[106]^

The hollow fiber assay is used as a preliminary step for the further study of compounds in xenograft models.^[107]^ In this assay, biocompatible hollow fibers made of poly(vinylidene fluoride) are developed with a size of 2 cm and an inner diameter of 1 mm, and incubated with tumor cells for 1 day;^[108]^ angiogenesis can be thereafter quantified by immunohistochemical staining of paraffin-embedded sections of hollow fibers. This assay has numerous advantages and provides the opportunity to identify the molecular mechanisms that play roles in angiogenesis; moreover, analogously to other implant assays, inflammation around the fibers may contribute to the interpretation of neoangiogenesis mechanisms.^[107]^

The quantification of angiogenesis is performed with invasive and noninvasive methods. In invasive techniques, angiogenesis is monitored with light or electron microscopy and histological tests in the sectioned tissues. The tissue sections are stained owing to endothelial cell antibodies or treated with intravascular markers, such as colloidal carbon, India ink, and radioactively-labeled red blood cells.^[109]^ Additionally, the discrimination of neovascularization and existing vessels can be performed with active endothelial cell selective antibodies.^[109]^ On the other hand, noninvasive techniques, such as dynamic MR scanning, functional CT, and PET scanning are reproducibly used to monitor angiogenesis in patients; however, the low spatial resolutions limits the use of these methods.^[69,70]^

#### 3.2.2 In Vitro Assays

Endothelial cell proliferation is analyzed based on cell number and cell cycle kinetics to reveal whether cells are activated towards vascularization.^[110]^ The net cell number is detected by simply counting using a hemocytometer under light microscope; however, this is time consuming and not sensitive; for this reason, electronic cell counters are preferred.^[88]^ On the other hand, colorimetric cell viability assays are appropriate candidates to quantify live endothelial cells, including water-soluble tetrazolium salt (WST)-1 or WST-8. In these assays, cells are exposed to tetra-zolium salts that are cleaved to formazan by mitochondrial dehydrogenase enzymes, and metabolic activity is deduced by the quantification of formazan products.^[111]^ The cell cycle distribution of endothelial cells is evaluated by staining intracellular deoxyribonucleic acid (DNA) with propidium iodide (PI), which specifically intercalates the DNA double helix. Therefore, the fluorescent signal intensity coming from PI indicates the phase of the cell cycle, and thus their proliferation tendency.^[112]^

Endothelial tip cells must migrate through the end points of vessels by crossing the laminin-rich basement membrane and collagen-rich extracellular matrix. In this situation, endothelial cells degrade the basement membrane and extracellular matrix by the overexpression of matrix metalloproteinase (MMP) pro-teases. Therefore, MMP production in endothelial cells is evaluated as a clue about possible tumor angiogenesis in tumor tissues.^[113]^ In the literature, an MMP detection assay named as zymogen assay has been elaborated to detect the MMP production profile in endothelial cells.^[114]^ As a low-cost technique, the zymogen assay provides basic information about the MMP expression profile of cells; however, it is a time-consuming and difficult technique for use in multiple experiments.^[114]^

Endothelial cell differentiation is referred to the generation of 3D tubule formation presenting tight junctions to maintain a strong tubule form. The differentiation ability of endothelial cells depends on the used matrix, including Matrigel, collagen, or fibrinogen.^[99]^ In another kind of assay, endothelial cells are cultured with stromal cells that empowered the generation of tubules thanks to their secreted matrix components without the necessity of adding extracellular matrix to the culture media.^[115]^

In the comparison of in vitro and in vivo assays involved in the evaluation of angiogenesis, some advantageous and disadvantageous aspects should be considered, before choosing the most appropriate tool. In in vitro assays, endothelial cell functions alone can be analyzed for their effects on angiogenesis, while in in vivo experiments, multiple cell types and other components are involved in the analysis, which can make more complex the evaluation of the direct role of endothelial cells. However, in vivo experiments provide an opportunity to follow the angiogenesis of cells in their natural environment; additionally, in vitro assays can provide detailed information about the stages of angiogenesis, including cell proliferation, migration, and generation of tubules, whereas in vivo assays provide information about angiogenesis at a whole. In addition, in vitro assays do not require technical proficiency, and are more cost-effective tools with respect to in vivo analyses. Angiogenesis can be also quantified in vitro more easily than in vivo experiments, albeit the results could not be sufficiently realistic.

### 3.3 Chemokine Detection in Metastasis and Angiogenesis Evaluation

In recent decades, a major question in metastasis research is why tumor cells preferentially metastasize to a particular organ and not to others. Mechanical reasons and organ-specific physiology are believed to be the determining factors in metastasis destination.^[116]^ Several studies have demonstrated that some chemotactic factors guide primary cancer cells to their destination.^[117]^ Chemokines, such as chemotactic cytokines from specific embryonic origins or from the immune system, appear by epigenetic modifications of primary tumor cells and regulate metastatic behavior.^[118,119]^ It is well known that embryonic cells have chemokine receptors that respond to ligands during organ development and tissue generation;^[120]^ tumor cells mimic the function of embryonic cells by producing chemokine receptors with specific epigenetic modifications established by histone deacetylations or by DNA methylations.^[121]^ Chemokine receptors provide cells with chemotactic responsiveness to chemokines in local and distant organ sites. One of the most interesting examples of chemokines is C-X-C M-motif chemokine receptor 4 (CXCR4) and its ligand C-X-C motif chemokine ligand 12 (CXCL12), which originally control the travel of germ cells to the gonads during the embryonic term.^[122]^ On the other hand, the correlation of CXCR4/CXCL12 with the metastasis of melanoma and breast cancers through lymph nodes is strongly supported by evidence.^[123]^ The chemokine receptor CXCR4 is detected in microdissected melanoma tissues, and increased CXCL12 chemokine is detected in the lung, bone, and lymph nodes of patients. In other studies, it has been demonstrated that CXCR4 receptors also play significant roles in breast and colorectal cancers.^[124,125]^ In another example, C-X-C chemokine receptor type 7 (CXCR7) was found to be required for immune cells such as dendritic cells (DCs), Langerhans cells (LCs), T cells, and natural killer cells bearing CXCR7 to enter lymph nodes that contain the chemokine ligand C-X-C chemokine ligand 21 (CXCL21).^[126–128]^

Recently, it has been shown that breast cancer cells and cutaneous primary and metastatic melanoma cells express C-C chemokine receptor type 7 (CCR7) in a manner that facilitates the metastasis of these cells from the primary site to the lymph nodes.^[129,130]^ Moreover, organ-specific metastasis of other cancers has been examined by investigating the response of receptors to CXCR4 ligand expression in lung, bone and lymph nodes,^[131]^ CCR7 in lymph nodes,^[129]^ C-X3-C motif chemokine receptor 1 (CX3CR1) in brain,^[132]^ C-C chemokine receptor type 5 (CCR5) and C-X-C motif chemokine receptor 2 (CXCR2) in lung, liver, vessel endothelial cells and bone,^[133,134]^ and C-C chemokine receptor type 9 (CCR9) in liver and small bowel.^[135,136]^

In angiogenesis, chemokines play a significant role in the stimulation of both vascular endothelial cell proliferation and in capillary tube formation.^[137]^ Among all chemokines, C-X-C motif chemokine ligand 8 (CXCL8) is the most studied proangiogenic molecule that supports the formation of new blood vessels.^[138]^ However, CXCL8-dependent angiogenesis stimulation in the absence of an inflammatory reaction suggests that CXCL8 works independently from proinflammatory reactions.^[139]^ In addition, a number of studies have shown a correlation between CXCL8 overexpression, measured by serum levels, and angiogenesis, especially in prostate and breast cancers.^[140,141]^ It has also been demonstrated that CXCL8 shows chemotactic effects directly on C-X-C motif chemokine receptor 1 (CXCR1)- and CXCR2-receptor-expressing endothelial cells by upregulating MMP-2 and MMP-9 factors, which are required for endothelial cell migration, organization, and angiogenesis.^[142]^ In addition to CXCL8, an elevated level of CXCL5 in serum is correlated with angiogenesis in nonsmall cell lung cancer;^[143]^ in addition, high CXCL1 and CXCL3 expression in plasma correlates with renal cell cancer, and high CXCL2 levels were detected in endothelial cells within tumor biopsies.^[144]^

Chemokines play thus an important role in tumorigenic expansion, and contribute to regulate the metastatic and angiogenic behavior of tumors. The detection of chemokine or chemokine receptor production by tumor cells in metastasis or angiogenesis is made possible by quantitative and qualitative molecular analyses, including real-time reverse transcription-polymerase chain reaction (qRT–PCR), immunohistochemistry, and Western blotting.^[145]^ In addition, cell migration assays are conducted to assess the effects of altered chemokine receptor expression on cell motility;^[85]^ finally, methylation-specific PCR can be performed to analyze promoter methylation of chemokine receptors.^[146]^

## 4 Cancer Nanotechnology

For cancer therapy, effective and commonly used methods comprise surgery, chemotherapy, and radiotherapy, despite their side effects on normal cells. In addition, gene therapy, immunotherapy, hormone-based therapy, and stem cell therapy have been introduced to minimize the chance of relapse in cancer patients, and can be used in combination with traditional treatments.^[147]^ On the other hand, nanotechnology suggests the use of biocompatible and biodegradable systems that can increase the bioavailability and the concentration of conventional drugs at the desired site, as well as tailor release profile in the tumor area.^[148]^

Cancer nanotheranostic includes injectable drug-loaded nanovectors such as liposomes, MRI agents, and novel nanoparticle-based methods for the detection of DNA and protein.^[149]^ The clinical success of nanoparticles is related to their safety profile, stability in circulation, bioavailability at the tumor site, and ability to cross physiological barriers and reach the disease site.^[7,150]^ Imaging and diagnostics are critical in the treatment of metastatic tumors, since precise tumor localization is very important, for example, in targeted radiotherapy. Nanomaterials for cancer therapy can vary from carriers of drugs or biomacromolecules (small interfering RNA or proteins) to vehicles for hyperthermia generation. The targeting of nanomaterials to tumor sites might occur in two steps: the primary targeting of nanomaterials to the specific organs or organs where metastasis has already occurred, and the secondary targeting that delivers nanomaterials to either cancer cells or specific subcellular locations in tumor cells.^[7]^

One of the bottlenecks of traditional methods in cancer therapy is the inability to deliver an adequate quantity of cancer drugs to the tumor area without side effects. However, the size (1–100 nm) greatly increases the surface area compared to bulk materials, and the unique mechanical, magnetic, optical, and electronic features of nanomaterials suggest their use in a wide range of applications, including imaging and drug delivery.^[151]^ In recent years, studies in cancer nanotechnology have increased dramatically since the first approval of a liposomal formulation (Doxil) for ovarian cancer and Kaposi’s sarcoma by the FDA.^[5]^ The recent approvals of nanopharmaceuticals, such as patisiran (RNAi therapeutic),^[152]^ NBTXR3 (a radio-enhancing nanoparticle),^[153]^ and Vyxeos (a mixture of two drugs at a synergistic ratio)^[154]^ demonstrate the importance of nanomaterial-based strategies in the development of novel cancer treatment modalities.

Comprehensive studies of nanomaterials are reported in the literature, indicating their importance also in cancer metastasis and angiogenesis;^[7,155,156]^ nanomaterial-based strategies will be highlighted in this regard, with special attention to inorganic, polymeric, and lipid-based nanomaterials to counteract cancer metastasis and neoangiogenesis.

### 4.1 Inorganic Nanomaterials

Inorganic nanomaterials have attracted significant interest in the last two decades due to their biocompatibility, stability, inertness, facile preparation, wide surface conjugation chemistry, ease of functionalization, and unique material- and size-dependent physicochemical properties. Inorganic nanomaterials, including AuNPs, magnetic nanoparticles (MNPs), quantum dots (QDs), silica nanoparticles, and carbon-based nanoparticles, have been designed and studied for the diagnosis and treatment of several diseases, and are commonly used in cancer therapy, particularly in hyperthermia, photodynamic therapy, and in drug delivery applications.^[157–159]^

#### 4.1.1 AuNPs

Among inorganic nanomaterials, AuNPs have been widely studied for biomedical applications since several years.^[160]^ Unmodified AuNPs were studied to demonstrate the inhibitory effect on tumor growth and metastatic nodules in ovarian carcinoma in vitro (A2780, OVCAR5, SKOV3-ip, and ovarian surface epithelial -OSE-cell lines) and in vivo.^[161]^ The results showed that AuNPs reduced the secretion of a number of proteins involved in EMT, upregulated E-cadherin, and downregulated of N-cadherin, vi-mentin, and Snail. In addition, an increase in Serpin E1 was observed, which is related to tumor angiogenesis and to reduction of tumor blood vessels in vivo. The overall data showed the reversal of EMT and the inhibition of mitogen-activated protein kinase signaling, which has a crucial role in tumor proliferation.^[161^] It has also been reported that the nuclear targeting of AuNPs via three ligands, methoxy(polyethylene glycol) thiol (PEG-thiol), RGD (RGDRGDRGDRGDPGC), and nuclear localization signal (NLS) peptides, inhibits the cell migration and invasion of ovarian cancer cells by increasing the expression of lamin A/C protein and thus nuclear stiffness, which is related to decreased cell migration.^[162]^ Yang et al. reported the effect of AuNPs in a prostate cancer cell line (PC3) compared to human dermal fibroblasts (HDFs).^[163]^ Decreased cell migration was observed for prostate carcinoma cells, while HDF migration was related to the surface charge and shape of AuNPs. They suggested that protein corona and the inherent cell migration mechanism of cancer or fibroblast cells may be the reasons for observed results.^[163]^

Ali et al. developed Arg–Gly–Asp (RGD) peptide-functionalized gold nanorods (RGD-AuNRs) to inhibit human oral squamous cell carcinoma (HSC-3) migration by targeting integrins.^[164]^ Additionally, 808 nm near-infrared (NIR) light was exploited to produce heat through photothermal therapy (PTT). The analyses showed the downregulation of integrins and broad perturbations in actin-, microtubule-, Rho GTPase-, and kinase-related pathways, which cause decreased cell migration. Moreover, PTT impaired migration abilities, enhancing cytoskeletal remodeling.^[164]^ In addition to this study, Tay et al. investigated the cell migration mechanism on human squamous cell carcinoma (TR146 cells) after inorganic nanoparticles (titanium dioxide, silicon dioxide, and hydroxyapatite) treatment. The results showed a decreased cell sheet migration because of destabilization of microtubule network; this leads to a significant change in magnitude and spatiotemporal distribution of cell traction, which reduces cell motility.^[165]^

In another study, Zhou et al. developed bovine serum albumin (BSA)- and *m*PEG-coated and immunoadjuvant imiquimod (R837)-loaded gold nanorods (mPEG-GNRs@BSA/R837) for the treatment of metastatic melanoma, by combining PTT and immunotherapy.^[166]^ Under NIR stimulation, the prepared nanosystem triggered a strong immune response in vitro (on B16-F10 cells) due to increasing levels of tumor necrosis factor (TNF)-α, interleukin-6 (IL-6), and interleukin-12 (IL-12) after treatment. In addition, in vivo experiments on C57/BL6 mice showed the effective inhibition of tumor metastasis in lung through immunotherapy.^[166]^

In another study, serum protein-coated gold nanorods (AuNRs) were used in three different metastatic cancer cell lines: PC3, MDA-MB-231 (human breast cancer cell line), and B16F10 (mouse melanoma cell line).^[167]^ It was reported that a low concentration (50 × 10^-6^ M) did not significantly affect cell proliferation and viability; however, it effectively inhibited in vitro cell migration and invasion by downregulating the expression of energy generation-related genes, such as mitochondrial oxidative phosphorylation (OXPHOS) and glycolysis. The impairment of these genes reduces adenosine triphosphate synthesis, leading to decreased filamentous actin (F-actin) in cytoskeleton, which has an important role in cell migration and invasion. Furthermore, the inhibitory effect of AuNRs was confirmed in vivo in mouse models investigating the metastatic ability of MDA-MB-231 cells, and a significant reduction in metastatic nodules was observed.^[167]^

Low molecular weight heparin is a polymer preferentially used in conjugation with metal nanoparticles as antiangiogenic agent in cancer treatment;^[168]^ effects of heparin-conjugated silver and gold nanoparticles were for example examined using a chicken chorioallantoic membrane (CAM) model in vivo. A significant inhibition of fibroblast growth factor (FGF)-induced angiogenesis was found;^[169]^ since proangiogenic factors, such as interleukin-8 (IL-8) and FGF-2, binds to heparin sulfate receptors located at endothelial cell surface, they also show high affinity to the heparin molecules, thus down-regulating angiogenesis in vascular endothelial cells.^[170]^

#### 4.1.2 MNPs

Using MNPs in cancer therapy provides the possibility of drug accumulation into previously determined area, since MNPs can be manipulated by using static magnetic fields; moreover, they are also used for local hyperthermia since they can generate heating via an alternating magnetic field.^[171]^

In one example, magnetic hyperthermia therapy (MHT) was combined with checkpoint blockade immunotherapy to show the inhibitory effect of superparamagnetic CoFe_2_O_4_@MnFe_2_O_4_ nanoparticles in both primary and metastatic tumors.^[172]^ During therapy, several tumor-associated antigens were produced, leading to effective immunotherapy for metastatic tumors in 4T1 tumors in BALB/c mice by promoting the activation of dendritic and cytotoxic T cells.^[172]^

In another study, the effect of cobalt ferrite nanoparticles features on MHT was comparatively studied in two murine models (breast 4T1 cells and colon CT26 cells) in vitro and in vivo.^[173]^ In vitro studies showed that MHT killing capacity increased in both cell lines, being CT26 cells more sensitive to heat because of the activation of antioxidant proteins and low expression of heat shock proteins (HSPs). Furthermore, complete 4T1 primary clearance and more effective prevention of metastasis were observed in high-temperature MHT (>47 °C) with respect to surgical extraction.^[173]^

In another study, polyethylenimine (PEI)-coated superparam-agnetic iron oxide nanoparticles (SPIONs) were shown to impair angiogenesis in vitro and in vivo.^[174]^ The effects of PEI–SPIONs on murine and human endothelial cells were evaluated using functional angiogenesis and gene profiling assays, and PEI–SPIONs downregulated the proangiogenic cytokine transforming growth factor beta (TGF-β). Furthermore, it was observed that PEI–SPIONs decreased the expression of *Rac1, Akt1, MMP9,* and *MMP14* genes associated with cell migration. In vitro endothelial tube formation assay showed a decreased tube formation due to increasing levels of cluster of differentiation 54 (CD54) integrin expression. Besides, PEI–SPIONs altered actin polymerization (reduced Src and cortactin phosphorylation). In vivo experiments on MDA-MB-231 xenograft tumors in nude mice showed reduced blood vessels and promotion of macrophages infiltration, suggesting potential use of SPIONs as antiangiogenic antitumoral agents.^[174]^

Xu et al. developed octagonal-shaped iron oxide nanoparticles, Octapod-30, to evaluate TAMs in pancreatic ductal adenocarcinoma, and to determine the effect of obesity on TAMs.^[175]^ TAMs are found in tumor environment and are responsible for tumor progression, metastasis, and angiogenesis. In vitro (using murine pancreatic ductal adenocarcinoma cells, -panc02-, and liver cells, -NCTC1469-) and in vivo experiments confirmed the biocompatibility of Octapod-30. After administration of Octapod-30, a higher amount of TAMs was found in tumor microenvironment of obese mice; this study suggests noninvasively in vivo detection of TAMs via MRI with high sensitivity and selectivity by exploiting Octapod-30.^[175]^

#### 4.1.3 QDs

QDs gained significant interest in the scientific community due to their high quantum yield, wide absorption with narrow and photoluminescence spectra, high resistance to photobleaching, remarkable resistance to photo- and chemical degradation, and higher molar extinction coefficients with respect to organic fluorophores.^[176]^ QD-based probes are generally used for in vivo imaging, anticancer drug/siRNA delivery, cancer diagnosis, and MRI.^[177]^

In a recent study, QDs were modified with asparagine– glycine–arginine (Asn–Gly–Arg, NGR) for binding to CD13 receptor, overexpressed in pancreatic cancer and related to tumor progression, metastasis, and angiogenesis.^[178]^ Gadolinium (Gd^3+^) was conjugated to the prepared nanoprobes in order to enhance MRI spatial and functional information. Decreased cell proliferation, invasion, and metastasis were found on pancreatic cell line (PANC-1 cells) due to the increased reactive oxygen species (ROS) and apoptosis.^[178]^

Roshini et al. investigated the antimetastatic properties of tangeretin-zinc oxide quantum dots (Tan-ZnO QDs). Tan is a natural polymethoxyflavonoid and is used as anticancer agent.^[179]^ Decreased cell proliferation and migration, regulation of apoptotic Bax and Bcl-2 proteins expression, cell cycle arrest in G2/M phase, and induced apoptosis and nuclear fragmentation were found in H358 metastatic lung cancer cells. Metastasis markers, MMP2 and MMP9, and angiogenesis marker, VEGF, were significantly downregulated. Furthermore, this system facilitated tracking during chemotherapy due to the luminescent properties of ZnO QDs.^[179]^

Since the early detection of tumor angiogenesis is important to avoid tumor spreading, Ag_2_S QDs were proposed as a new kind of fluorescent probe in the second NIR window (1000– 1350 nm) to observe angiogenesis occurring in 2–3 mm diameter tiny tumors.^[180]^ PEGylated Ag_2_S QDs were subcutaneously injected into the mice, and the lymph nodes and vessel anatomy were observed. The results showed that Ag_2_S QDs can be used to visualize real-time tumor angiogenesis and circulatory blood systems in vivo owing to their deep tissue penetration, high spatial and temporal resolution, and low autofluorescence. It is suggested that these nanoprobes might be used not only in surgical treatments, but also in screening antiangiogenic drugs.^[180]^

Recently, cancer immunotherapy that activates the patient’s immune system attracted great interest for either reduction of long-term cancer metastasis and recurrence.^[181]^ Liu et al. developed QD pulsed-dendritic cell (DC) vaccines for improved antitumor immunity, since DCs are essential for the activation of immune responses.^[182]^ QDs provide several functions, acting as fluorescence probes, nanocarriers for vaccine components, and as immunomodulatory adjuvants due to Nlrp3 (NLR family pyrin domain containing 3)-dependent inflammasome activation pathway. Lung metastasis decreased in treated B16-F10 tumor bearing C57BL/6j mice due to the polarization of TAMs caused by the loss of chemokine (C-C motif) ligand 3 (CCL3), suggesting the use of QD pulsed-DC vaccines for improved cancer immunotherapy.^[182]^

In another study, adult mesenchymal stem cells (MSCs) were used as nanoparticle vehicles of carboxylated CdSe/ZnS QDs in human breast cancer cells (MDA-MB-231), in human mammary epithelial cells (MCF-10A), and in SCID mice.^[183]^ First of all, the migration capacity of MSCs toward cancer cells was demonstrated in vitro and in vivo. Then, QDs-loaded MSCs were exploited for in vivo imaging of migration thanks to nanoparticle imaging properties; the results showed that QD-labeled MSCs were selectively located in tumor and in metastatic sites.^[183]^

#### 4.1.4 Silica Nanoparticles

Among inorganic nanomaterials, silica nanoparticles have plenty of useful features, such as versatile silane chemistry for surface modification, a hydrophilic surface favoring circulation in the bloodstream, and a low cost of production. In addition, the approval by the FDA of ultrasmall cancer-selective silica particles (≈7 nm diameter) developed in 2011 for the targeted molecular imaging of melanoma cancer further increased the interest in silica nanoparticles.^[184,185]^

Lipid-coated biodegradable hollow mesoporous silica nanoparticles (dHMSB) were prepared for the codelivery of three agents: doxorubicin (DOX) as a chemotherapeutic drug, all-trans retinoic acid (ATRA) as an immunosuppressive agent, and interleukin-2 (IL-2) as an immunotherapeutic agent for melanoma treatment.^[186]^ This treatment activates tumor-infiltrating T lymphocytes and natural killer cells (NK cells), promotes the secretion of cytokines such as interferon gamma (IFN-γ) and interleukin-12 (IL-12), and downregulates interleukin-10 (IL-10), TGF-β, and immunosuppressive myeloid-derived suppressor cells. Thus, this drug delivery system demonstrated not only a significant decrease in tumor growth and metastasis, yet also a favorable safety profile, as confirmed by the low levels of alanine aminotransferase, aspartate transaminase, and blood urea nitrogen, and no cardiac pathological change observed in vivo; also biodegradability was satisfactory, thanks to high surface area and large pores presenting thin pore walls.^[186]^

In another study, fluorescent silica nanoparticles (labeled with cyanine dyes, Cy3(+) and Cy5(+)) were studied as a labeling strategy to analyze tumor metastasis into the mineralized bone microenvironment.^[188]^ MDA-MB-231 breast cancer cells, as a 3D in vitro model and an in vivo model of bone metastasis, were used to demonstrate the simultaneous imaging of cells, bone marrow, and mineralized matrix in metastatic tumor cells. Unlike proteinbased labels, silica-based particles have an electron-dense core that enables detection by electron-based imaging techniques. In addition, their compatibility makes them suitable for in vitro and in vivo studies for fluorescence imaging techniques such as confocal, widefield, and light sheet microscopy.^[188]^

**Figure 4:**
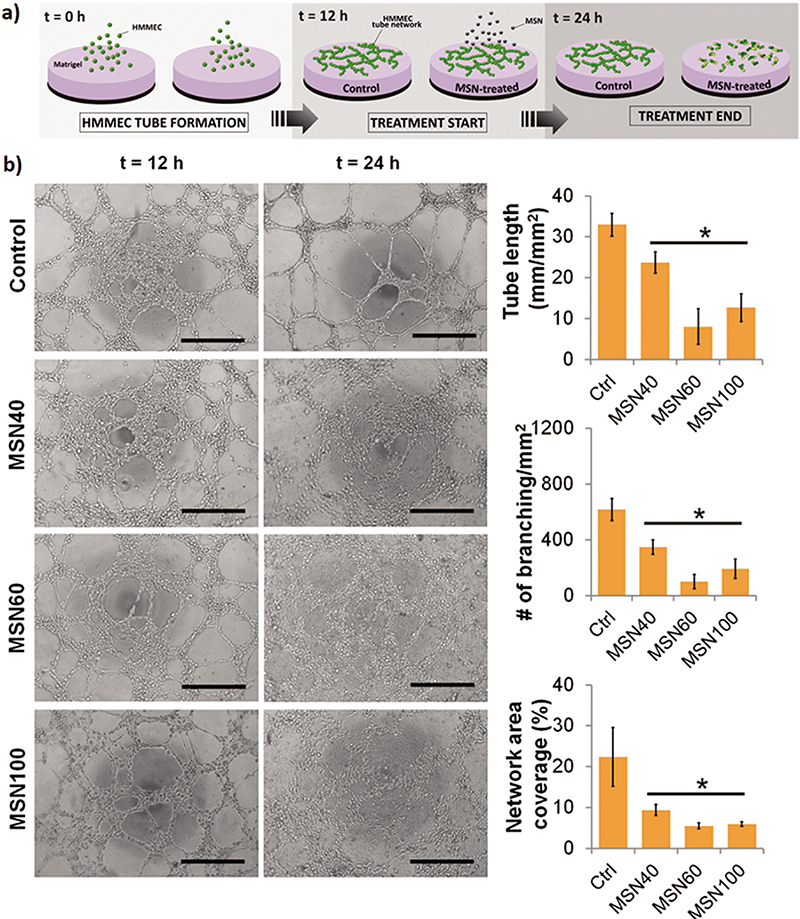
Disruption of the existing human mammary microvascular endothelial cells tubes in the presence of MSNs. a) Schematic representation of the tube formation assay. b) Phase contrast images and semiquantitative analysis (scale bar: 100 μm). Reproduced with permission.^[187]^ Copyright 2017, American Chemical Society.

Neoangiogenesis is strictly related to metastatic phenomena, as new sprouting vessels can be successfully exploited by cancer cells to travel until the blood stream; it is therefore clear that many strategies focus on the inhibition of angiogenic activity for successful metastatic cancer therapy.^[187]^ Setyawati et al. showed the size-dependent effect of mesoporous silica nanoparticles (MSNs) on tumor angiogenesis: Figure 4 shows the efficacy of MSNs on pre-existing blood vessel tubes. It has been demonstrated that the antiangiogenic activity of MSNs is derived from the generation of ROS, which interfere with the p53 tumor suppressor pathway. Activation of the p53 signaling pathway leads to the inhibition of cancer cell migration, invasion and proliferation.^[187]^

#### 4.1.5 Carbon-Based Nanomaterials

Carbon-based nanoparticles are largely preferred in drug delivery applications as carrier agents over other nanoparticles, owing to their, easy conjugation with other macromolecules.^[189]^ However, their relatively low biocompatibility and biodegradability must be considered before their biomedical application; on the other hand, a mild cytotoxicity could be advantageous for their utilization as therapeutic agents in cancer treatment.^[190,191]^

Multiwalled carbon nanotubes (MWNTs), C_60_ fullerenes, and graphite have been examined for their antiangiogenic effects concerning the binding affinity of angiogenesis activating receptors (VEGF and FGF2).^[192]^ According to collected results, these carbon-based nanoparticles showed antiangiogenic effects by inhibiting the proliferation of human umbilical vein endothelial cells (HUVECs) in in vitro tests.^[192]^ In another study, diamond nanoparticles, graphite nanoparticles, graphene nanosheets, MWNTs, and C_60_ fullerenes were comparatively evaluated for their angiogenic activity.^[193]^ Diamond nanoparticles and MWNTs showed excellent antiangiogenic effects, while fullerene instead stimulated blood vessel development in an in ovo chick embryo chorioallantoic membrane model. In addition, graphite nanoparticles and graphene did not show any significant effect. In detail, the protein expression profile of the cells indicated that proangiogenic proteins, including VEGF, were significantly inhibited in the samples exposed to the diamond nanoparticles, which encouraged evaluation of their versatile antiangiogenic effects.^[193]^

The antiangiogenic effects of diamond nanoparticles were evaluated in detail by Wierzbicki et al.^[194]^ Caveolin-1, a multifunctional protein and a major component of caveolar membranes that participates in the regulation of angiogenic signaling pathways in endothelial cells, was evaluated in diamond nanoparticle-exposed cells.^[195]^ Caveolin-1 inhibits the function of proangiogenic factors, including VEGF and the Akt and Stat3 signaling pathways, which are important in the development of new blood vessels.^[196]^ Two different types of carbon nanoparticles were evaluated comparatively: diamond nanoparticles and graphite nanoparticles of the same size (3–5 nm); 3D analysis of chorion membranes demonstrated that the diamond nanoparticles changed the intracellular distribution of cavolin-1 proteins, while graphite nanoparticles did not show any significant effects. This result is attributed to the downregulation of proangiogenic factors, including VEGF and Akt and Stat3 signaling pathways. In another study, it was revealed that diamond nanoparticles reduced angiogenesis by inhibiting VEGF receptor expression in endothelial cells.^[197]^

Carbon nanotubes have been used in many applications as drug carriers against cancer metastasis and angiogenesis^[198]^ for various drugs, including doxorubicin,^[199]^ carboplatin,^[200]^ and paclitaxel.^[201]^ In one study, MWNTs were functionalized with poly(acrylic acid) and decorated with magnetic nanoparticles (iron oxide, Fe_3_O_4_).^[202]^ This magnetic lymphatic targeting system was targeted to metastatic lymph nodes to deliver gemcitabine with high efficiency under the guidance of a magnetic field. The results are encouraging, as this method will enable a more effective means of targeting chemotherapeutic drugs at metastatic lymph nodes, and may provide an opportunity to treat cancers locally without significant systemic toxicity.^[202]^

MWNTs have been also exploited as carriers of tumor antigens along with different types of immunoadjuvants to antigen presenting cells, in order to stimulate immune system against tumor. MWNTs have been functionalized with cytosine-phosphate-guanine oligodeoxynucleotide (CpG) and anti-CD40 Ig (aCD40) as immunoadjuvants, together with the model antigen ovalbumin (OVA). The structure was evaluated in a standard metastatic tumor model of melanoma (B16F10 metastatic cells), but also on a lung pseudometastatic tumor model, consisting in OVA-B16F10-*luc* cells subcutaneously injected into C57BL/6 mice. OVA functionalization onto the CpG-conjugated MWNTs improved the CpG-mediated immune response, having efficient immune stimulation being found to be associated with the cytolytic activity of CD8^+^ T cells.^[203]^

### 4.2 Polymeric Nanomaterials

**Figure 5:**
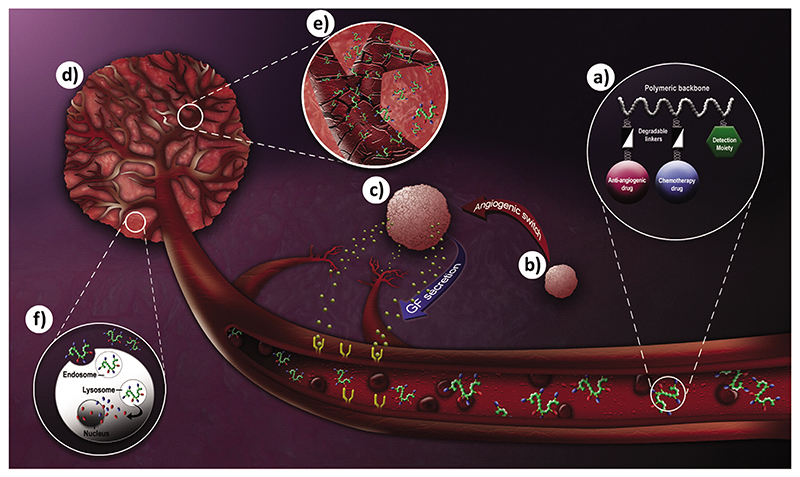
Schematic illustration of tumor vasculature targeting using polymer conjugates of antiangiogenic agents. a) Polymeric drug-delivery system composed of polymeric carrier, degradable linkers, an antiangiogenic drug, a chemotherapeutic drug, and a detection moiety; b) an angiogenic stimulating cells; c) an angiogenic stimulation induced by the secretion of vascular growth factors (VGF), encouraging the development of neovascularization; d) a well-established vascularized tumor; e) the EPR effect allowing the extravasation of polymer therapeutics through hyperpermeable tumor blood vessels and their accumulation at the tumor site; f) cellular uptake of the polymeric drug delivery system via endocytosis followed by drug release into the cell cytoplasm. Reproduced with permission.^[206]^ Copyright 2009, Elsevier.

Many drug delivery applications have been performed with polymeric nanoparticles basically developed from plain or conjugated natural or synthetic polymers, functionalized with drugs and targeting agents (Figure 5). In this regard, cationic polymers are widely used for drug delivery applications, because of their high cellular internalization ability through the negatively charged cell membranes. A well-designed polymeric drug delivery system also improves the half-life of drugs, providing the required time for their accumulation in the diseased sites.^[204]^ Moreover, the limited cellular uptake and weak stability of free drugs in blood circulation dramatically decrease their treatment efficiency, and the design of an efficient drug carrier system that is stable in blood circulation and can encapsulate a large quantity of drugs with a preferential targeting response is essential for successful cancer therapy.^[205]^

Tumor vessels have a smaller surface area than normal vessels; the high interstitial pressure, coupled with the reduced surface area, leads to serious impairment of the delivery of therapeutic agents into tumorigenic vessel cells via blood circulation. Therefore, small nanoparticles developed to an appropriate size for transport through the narrow intercellular openings (100– 780 nm) in tumorigenic vessel structures are extremely useful in drug delivery.^[206]^

#### 4.2.1 Natural Polymers

As a natural polymer, chitosan is widely exploited for drug delivery applications because of its positively charge, which presents a high affinity for negatively charged cell membranes and provides an opportunity for passive cell targeting.^[207]^ However, chitosan-based cancer therapeutic strategies are generally limited by its concentration-dependent cytotoxicity.^[208]^ Recently, it was found that chitosan nanoparticles, specifically development for chemotherapy drug administration, resulted in preferential accumulation in cancer cells rather than in healthy cells, most probably because of the EPR effect.^[209]^ Chitosan nanoparticles were also evaluated on human hepatocellular carcinoma cells in a mouse xenograft model, in which the chitosan nanoparticledependent tumor-reducing effect was explained by the antiangiogenic efficiency of the nanostructure, which correlated with VEGFR2 production and subsequent blockage of VEGF-induced endothelial cell activation.^[210]^

Chitosan nanoparticles are also preferentially chosen as safe and effective carriers for small interfering RNA (siRNA);^[211]^ as an example, chitosan (CS) nanoparticles were conjugated with hydrophilic PEG molecules, which have been reported to enhance CS stability in a biological environment,^[212]^ improving transfection efficiency of siRNA.^[213]^ The siRNA was designed as a complementary of surviving gene (prometastatic gene) RNA and targeted to 4T1 breast cancer cells for selective degradation; siRNA-loaded PEGylated chitosan nanoparticles were able to inhibit tumor growth and downregulate the metastasis of 4T1 tumor breast cancer cells.^[212]^

In another study, carboxymethyl chitosan (CMC) was instead exploited as immune stimulating agent against tumor angiogenesis. Obtained results indicated that CMC significantly inhibited the 2D and 3D migration behavior of HUVECs in vitro. Moreover, CMC significantly hindered the growth of mouse hep-atocarcinoma by stimulating the CD34 expression, that down regulates the VEGF and metalloproteinase-1 secretion.^[214]^

Heparin is a naturally occurring polysaccharide, that gained wide interest and extensive use in clinical applications, although its potential to cause development of heparin-induced thrombo-cytopaenia type 2 antibodies, that means the activation of immune response against heparin-platelet factor 4 complex.^[215]^ Its high hydrophilicity, due to the presence of negatively charged groups, such as sulfonyl, carboxyl, and hydroxyl within its structure limits its exploitation as drug delivery agent,^[216]^ and thus quite often modifications of heparin structure are envisaged to improve its pharmacokinetic profile. As an example, sodium deoxycholate (DOC)-conjugated heparin nanoparticles have been tested on athymic BALB/c-nu/nu female nude mice xenograft models.^[217]^ Results demonstrated that the nanostructures can specifically target tumor and tumor vasculature, with minimal side effects. Promisingly, high antitumor activity and selective toxicity was found, achieving 34% tumor inhibition with respect to controls. The results are also consistent with an angiogenesis inhibition, showing a less detectable tumor vasculature after DOC-heparin treatment.^[217]^

Low molecular weight heparin (LMWH) represents instead an alternative avoid of many of the afore-mentioned heparin limitations.^[218]^ LMWH-based nanoparticles have been proposed as antiangiogenic structure because of their high affinity to growth factor receptors, such as VEGF and bFGF,^[219]^ while the conjugation with cholesterol (LHC) offered the opportunity to develop nanoparticles characterized by hydrophilic shell and cholesterol-based hydrophobic core structure.^[220]^ The presence of cholesterol has been exploited to obtain complexes with doxorubicin, suitable for intravenous direction; in vivo results showed that DOX/LHC nanoparticles demonstrate a stronger antimetastatic and antiangiogenic effect with respect to plain DOX. A synergic effect of DOX and LMWH has been supposed to be at the base of the observed effects; a further positive outcome has been represented by an increased blood circulation time: 6.12 h versus 1.53 h of the plain DOX.^[220]^

Hyaluronic acid (HA), as a linear polysaccharide, has been extensively evaluated in biomedical studies because it is a biocompatible, biodegradable, water soluble, and nonimmunogenic polymer.^[221]^ Among chemical derivatives of HA, C-6 hydroxyl group sulfonation of N-acetyl glucosamine repeats showed high affinity to VEGF receptors located on the vascular endothelial cell membrane.^[222]^ VEGF also regulates the tumorigenic angiogenesis mechanism in endothelial cells with its two different isoforms, angiogenic (VEGF_165a_) and antiangiogenic (VEGF_165b_) receptors. The sulfonated version of hyaluronic acid has a selective affinity to VEGF_165b_, in contrast to VEGF_165a_.^[223]^ By exploiting this mechanism, C-6 OH-sulfated HA nanoparticles have been found to inhibit HMVEC proliferation, demonstrating that the modification of HA through C-6 OH group sulfonation may be a promising strategy for the treatment of tumor angiogenesis.^[222]^

On the other hand, researchers have indicated that the different molecular weight/size ratio of HA shows opposite effects on tumor growth. Low molecular weight HA induces tumor growth, whereas high molecular weight HA shows preventive effects on tumor metastasis.^[225]^

**Figure 6:**
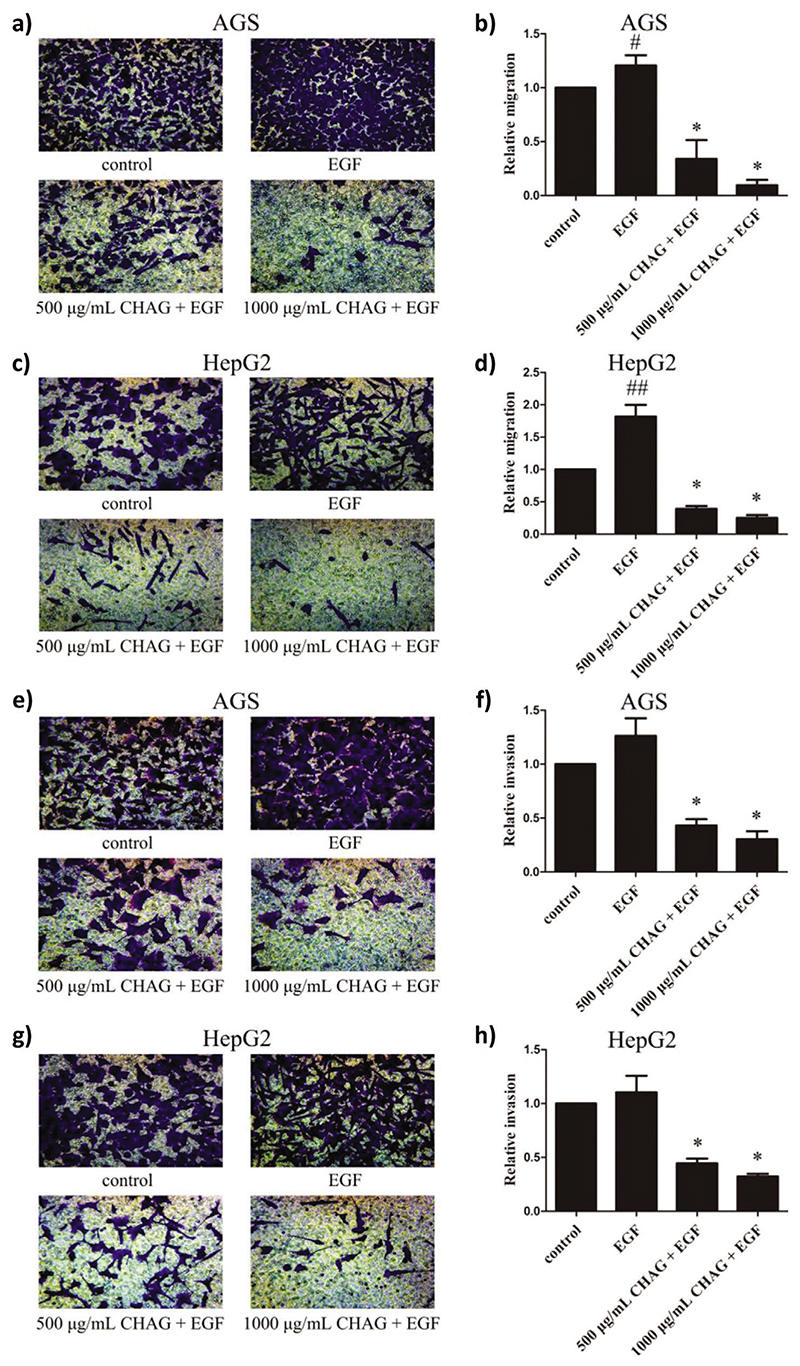
Inhibitory effects of CHAG on the migration and invasion of gastric and hepatic cancer cells. a–d) Migration activity of AGS and HepG2 cells. e–h) Invasion activity of AGS and HepG2 cells. The data shown are the means ± SD from 5 independent experiments, each performed in duplicate. (#*p* 0< 0.05, ## *p* < 0.01, compared with the control group; *p < 0.01, compared with the EGF group). Reproduced under the terms of the CCA 3.0 International Licence.^[224]^ Copyright 2016, Oncotarget.

It is already known that low molecular weight HA has affinity for cell proliferation receptors, including cluster of differentiation 44 (CD44) and HA receptor (RHAMM), which induce tumor growth.^[226,227]^ Thus, low molecular weight HA is known as a tumor marker, in contrast to high molecular weight HA.^[228]^ Accordingly, the possible antimetastatic effects of high molecular weight HA (cross-linked HA gel -CHAG-) were evaluated comprehensively to reveal the underlying molecular mechanisms.^[224]^ The results indicated that CHAG significantly reduced the migration and invasion of a gastric cancer cell line (AGS) and of a hepatic cancer cell line (HepG2), as shown in Figure 6. In detail, the CHAG-exposed protein expression profile of AGS and HepG2 cells indicated that integrin expression is downregulated by CHAG, as well as by the inhibition of the EGF-induced activation of EGFR- and VEGF-induced phosphorylation/activation of VEGFR-2. The inhibited EGF expression in the cells leads to the downregulation of metastatic inducer MMP receptors. Based on these results, CHAG was found to be a blocking agent of tumor metastasis and an efficient tumor progression inhibitor.^[224]^

#### 4.2.2 Synthetic Polymers

N-(2-hydroxypropyl) methacrylamide -HPMA-hydrophilic copolymers are intensively used for drug delivery applications, since they have a biocompatible and nonimmunogenic nature. Moreover, they have a tumor accumulation tendency that makes them suitable for antitumor and antiangiogenic applications.^[229]^ Especially in the systemic administration of severely cytotoxic chemotherapeutic drugs, HPMA conjugates provide an opportunity for biocompatible carriers until the drugs are removed from the polymers by cleavage in the target area.^[230,231]^ Recently, numerous HPMA-based therapeutic administrations have been investigated, and successful outcomes have been obtained with reduced side effects and preferential target region accumulation.^[232]^ In a drug delivery application, HPMA was conjugated with an antiangiogenic cytotoxic drug (TNP-470) named caplostatin and with aminobisphosphonate alendronate (ALN), resulting more stable in the blood circulation than free TNP-470 and ALN.^[233]^ Capsostatin and ALN molecules were bound to HPMA by a NH_2_ end groups including a peptide sequence (MA-Gly-Gly-Pro-Nle); as soon as caplostatin is internalized by the cells, the peptide sequence is cleaved by cathepsine K, and the drug is released. TNP-470 and ALN, as tumor angiogenesis reducing agents, prevent the establishment of neovascularizations; the conjugate inhibited human osteosarcoma growth in SCID mice by 96%, while a 45% reduction was found in free ALN plus TNP-470 treatment.^[233]^

In another study, performed by HPMA-ALN-TNP-470 administration, a new concept of combined therapeutic polymer design was shown, to target both tumor epithelial and vascular endothelial cells of bone metastases and calcified neoplasms,^[234]^ since ALN shows affinity to bone minerals, and its conjugation with caplostatin provides active targeting to calcified tissues. VEGF-induced vascular hyperpermeability was remarkably reduced by ≈92%, whereas osteosarcoma was inhibited by up to 96% in mice.^[234]^

In another study with HPMA, a caplostatin-based drug delivery strategy was modified with a chemotherapeutic agent, paclitaxel (PTX), in order to develop targeted treatment of prostate and breast cancers.^[235]^ Despite its strong antimetastatic and antiangiogenic effects in prostate cancer, PTX has a significant toxicity toward the neuronal and hematological systems. In this concern, conjugation of PTX into the caplostatin structure plays a very important role in reducing systemic toxicity.^[235]^

*β*-poly(L-malic acid) -PMLA-is another polymer analogously exploited. The abundant carboxyl groups in its structure provide an opportunity to develop multifunctional drug delivery systems;^[236]^ as an example, PMLA can be conjugated with pH-sensitive membrane disrupting units, polyethylene glycol (PEG), and cell-penetrating peptides.^[237]^ The whole structure (named as polycefin) has been conjugated with 2,3-dimethylmaleic anhydride (DMMA) (PMLA–PEG–TAT–DMMA), that provides pH responsive drug release.^[238]^ The PMLA–PEG–TAT–DMMA nanocomplex cellular uptake was comparatively investigated at pH 7.4 and 6.8, mimicking, respectively, healthy and cancer extracellular matrix pH values. The internalization of the nanocomplex at pH 6.8 was about 20-fold higher with respect to the internalization at pH 7.4. According to these results, it has been supposed that the hydrolysis of DMMA caused the charge reversal of the PEG layer, resulting into the exposure of the positively charged TAT-conjugated polymeric micelles (PMLA–PEI–DOX– TAT), responsible of an enhanced cell internalization.^[238]^

In another study, the polycefin structure was conjugated with transferrin to target angiogenesis and metastasis originating in glioblastoma. Polymeric prodrugs accumulate selectively in tumor tissue by the EPR effect and receptor-mediated endocytosis.^[239]^ The variability of the conjugated molecules in size and solubility, as well as the wide range of hydrophobic and hydrophilic drugs, indicates the highly convenient composition of PMLA-conjugated drug delivery systems for several applications.

Poly(glutamic acid) -PGA-is another good candidate for drug delivery applications, being a water-soluble, biocompatible, and biodegradable polymer.^[206]^ As an example, PGA conjugated with PTX (PGA–PTX) and with cyclic RGD peptidomimetic ((RGDfK)_2_) as targeting agent improved the effects of the plain drug,^[240]^ by inhibiting the α_v_β_3_ expression in endothelial cells and thus, as a consequence, by reducing their proliferation and migration; the final outcome is the arrest of capillary-like tube formation and the inhibition of endothelial cell attachment to fibrinogen.^[240]^

As an improved version of plain PGA-drug conjugates, PEG-conjugated PGA nanoparticles have been explored in a novel 3D model mimicking an infiltrated lymph node, on which antimetastatic effect of drug-loaded PGA–PEG nanoparticles were evaluated.^[241]^ Following the administration of nanoparticles, their selective interaction with the tumor cells was observed, and collected results provided necessary evidence to design a PGA– PEG-based drug delivery system to treat metastatic cells accumulated in lymph nodes.^[241]^

As one of the first FDA-approved polymers, poly(lactic-*co*-glycolic acid) -PLGA-is intensely studied for biomedical applications in human trials. It has been exploited for many drug delivery applications, including hormone-, cytokine-, drug-, and vaccine-based therapeutics.^[242]^ Moreover, depending on the desired drug release profile, the half-life of the PLGA polymers can be changed from some hours to weeks by modifying the monomeric composition. In addition, PLGA also has a quite flexible structure that can be adapted to meet the requirements of many active moieties to target tumor vasculature.^[243]^ As an example, PLGA has been used for local administration of drugs in place of systemic administration.^[244]^ Implantable nanoparticles have been obtained by PLGA conjugated with temozolomide (TMZ) to induce cytotoxic effect against C6 brain glioblastoma cells. Moreover, PLGA–TMZ microparticles were conjugated with vatalanib, as antiangiogenic agent, that has also showed antitumor activity in malignant gliomas. The combinational treatment demonstrated a significant decrease in cell proliferation, an increment of apoptosis, and a lower microvessel density within the glioma tumors.^[244]^

Poly(ethylene glycol) -PEG-is a nonbiodegradable, highly flexible polymer developed from linearly ordered ethylene monomers. In addition, it has a water and organic solvent soluble structure as well as biocompatibility.^[245]^ Its conjugation with active compounds, including proteins, peptides, hormones, and vaccines, is termed PEGylation and is generally performed by the covalent binding of one or more PEG molecules to the compounds. PEGylation is a commonly used technique that enhances the plasma half-life of compounds before their renal clearance from organisms,^[246]^ improving their pharmacokinetic profile. Moreover, the PEGylation of drug molecules provides macromolecular prodrug structures that limit their side effects by reducing systemic cytotoxicity.^[247]^ All this considered, PEG is a good candidate for delivery of antiangiogenic drugs by both passive targeting exploiting the EPR effect and by active targeting exploiting ad hoc functionalization.^[248]^

A very interesting study envisioned PLGA-*b*-PEG nanoparticles decorated with immunostimulant CpG-oligodeoxynucleotides (ODN) and functionalized with AuNPs for immune stimulation and PDT.^[249]^ Following the treatment of mouse bone marrow derived dendritic cells, the combination of PDT with a synergistic immunostimulant in a single polymer-based NP system showed a significant immune response, which can be even exploited for the treatment of metastatic cancer.^[214]^ Results indicated improvement in terms of immune stimulation, as evidenced by the increase in the level of proinflammatory/Th1-biased cytokines IL-2, IL-6, IL-12, and TNF-α, and minimal effects on immunosuppressants, such as IL-10.^[249]^

### 4.3 Lipid-Based Nanomaterials

In the last two decades, lipid-based nanomaterials including liposomes, solid lipid nanoparticles (SLNs), and nanostructured lipid carriers (NLCs), have attracted significant interest in cancer therapy owing to their low or no toxicity, controlled release and prolonged half-life circulation, and ability to entrap both hydrophobic and hydrophilic molecules.^[250,251]^ Lipid-based nanomaterials can be chemically modified (such as using PEG) to avoid the immune system or to enhance drug solubility, and can be prepared as a pH-sensitive formulation to release the drug in an acidic environment. Furthermore, they can be targeted through antibodies to enable recognition by tumor cells.^[251]^ After the first approval of a liposomal formulation (Doxil) by the FDA, a number of studies have been reported using lipid-based nanomaterials, and here we will focus on liposome-, SLN-, and NLC-based strategies to counteract cancer metastasis and angiogenesis.

#### 4.3.1 Liposomes

Liposomes are spherical nanosized vesicles usually composed by cholesterol and phospholipids. Their properties may vary depending on their lipid composition, size, surface charge, and preparation methods.^[252]^ Due to their high biocompatibility, biodegradability, and ability to transport both hydrophilic and hydrophobic drugs, they are commonly used as targeting agents in cancer therapy.

De et al. designed phosphatidylserine (PS)-targeted cationic liposomes alone (phosphatidylcholine–stearylamine, PC–SA) and in combination with DOX (DOX–PC–SA) to inhibit solid melanoma tumors and lung metastasis.^[253]^ Since the designed liposomes have their own anticancer activity owing to the specificity of surface-exposed PS, the combined therapy with DOX showed superior results inducing the upregulation of IFN-γ, IL-2, IL-12, andTNF-α. Furthermore, the upregulation of concanavalin A (ConA)-specific Th1 cytokines leads to the promotion of T cell immune activity, suggesting that this system not only destroys cancer cells, yet also activates the immune system against lung metastasis.^[253]^

To address the great obstacles at the base of the delivery of therapeutics to bone, Zhao et al. designed a glutamic oligopeptide (for bone affinity)-RGD peptide (for specific tumor recognition)-derived liposomal drug delivery system to improve the distribution of PTX in bone metastases of breast cancer.^[254]^ Both in vitro (using the MDA-MB-231 cell line) and in vivo (using Balb/c nu mice bearing MDA-MB-231 tumors) studies demonstrated superior targeting ability to hydroxyapatite receptor, which is a main component in bone tissue, and favored the accumulation of PTX in bone metastases due to the synergistic effect of dual targeting-mediated endocytosis.^[254]^

A dual complementary targeting was also exploited in DOX-encapsulating liposomes developed to target and recognize triplenegative breast cancer (TNBC) cells at primary and metastatic sites.^[255]^ Unlike conventional targeted drug delivery systems, which comprise a single ligand for targeting, in this study antibodies against intercellular adhesion molecule-1 (ICAM1) and epithelial growth factor receptor (EGFR) were exploited. The results of in vitro studies using three human TNBC cell lines (MDA-MB-231, MDA-MB-436, and MDA-MB-157) and one human non-neoplastic mammary epithelial cell line (MCF10A) showed increased ligand-receptor interaction, internalization via endocytosis, and therapeutic effects of the lipid system via simultaneous blockage of the EGFR and ICAM1 signaling cascades. TNBC lung metastases were moreover examined in tumorbearing mice by in vivo bioluminescence imaging, and an inhibition of orthotopic and lung metastasis was confirmed/^2551^

In another study, dual delivery liposomes loaded with immunosuppressive (indoximod -IND-) and chemotherapeutic (DOX) drug were developed for immunogenic cell death.^[256]^ IND inhibits the IDO-1 pathway,^[257]^ and the proposed liposomes showed significant enhancement in terms of immune response in either primary and metastatic tumor sites with respect to only DOX and DOX-loaded liposome. In addition, the combination of programmed death-1 (PD-1) blocking antibodies demonstrated strong lung metastasis reduction in an orthotopic 4T1 tumor model.^[256]^

In another example, pigment epithelium-derived factor (PEDF)–DNA-loaded liposomes were fabricated for cancer gene therapy against metastatic colorectal cancer.^[258]^ PEDF is known as an antiangiogenic and proapoptotic molecule; however, it presents some challenges due to the lack of efficient delivery. Therefore, PEDF–DNA-loaded liposomes were further modified with iRGD peptide for efficient targeting of pulmonary metastases of colorectal cancer; the results on a mouse colon cancer cell line (CT26) and on human umbilical vein endothelial cells showed inhibition of migration and invasion and the induction of apoptosis. In vivo studies also demonstrated strong inhibition of metastatic tumor nodules in the lung, with a prolonged survival time.^[258]^

The degradation of the ECM induced by MMPs affects the tumor microenvironment and can start the cascade for cancer metastasis. Lyu et al. prepared lysolipid-containing thermosensitive liposomes containing marimastat (MATT), an MMP inhibitor, to inhibit the expression and activity of MMPs in the treatment of breast cancer metastasis.^[259]^ A 20-fold decrease in tumor growth with enhanced accumulation was observed in 4T1 tumor-bearing mice due to the downregulation of *MMP-2* and *MMP-9* gene expression in vivo. Furthermore, a sevenfold reduction in metastatic nodules in the lung and a 6-fold decrease in the number of microvessels in the tumor site were found, demonstrating the antimetastatic and antiangiogenic effects of the liposomes.^[259]^

PEG-modified liposomes have been proposed for prolonged blood circulation properties and presenting improved targeting due to EPR effect;^[260]^ the addition of vascular cell targeting peptide (APRPG) decoration induced specific internalization by VEGF-stimulated HUVECs. This study indicates a promising strategy for the delivery of liposomes to tumor vessels with enhanced passive targeting through the EPR effect by PEGylation, as well as active targeting to the tumor vasculature by conjugation with the targeting APRPG peptide.^[260]^

#### 4.3.2 SLNs

SLNs consist of solid lipids and surfactants to improve their stability in biological media. The selection of solid lipids and surfactants affects size, stability, drug loading, and release. Furthermore, both hydrophobic and hydrophilic drugs may be entrapped in SLNs depending on the preparation method.^[261]^

Docetaxel-loaded SLNs (DTX–SLNs) were investigated in metastatic breast tumors in vitro using murine breast adenocarcinoma (4T1), human breast cancer (MCF7), and murine embryo fibroblast (NIH-3T3) cells and in vivo using 4T1-bearing BALB/c mice.^[262]^ It has been reported that DTX–SLNs induce apoptosis and microtubule damage, and decrease IL-6 production, B-cell lymphoma 2 (BCL-2) and Ki-67 expression, and tumor cell proliferation, leading to the inhibition of tumor progression and prevention of lung metastasis.^[262]^

In another study, PTX-loaded SLNs modified with Tyr-3-octreotide (TOC), a known ligand for somatostatin receptors overexpressed in glioma, were used for antiangiogenic and antiglioma therapy.^[263]^ The prepared lipid nanoparticles (PSMs) induced apoptosis in C6 glioma cells; moreover, a tube formation assay and CD31 staining showed relevant antiangiogenic activity of PSMs, both in vitro and in vivo.^[263]^

**Figure 7:**
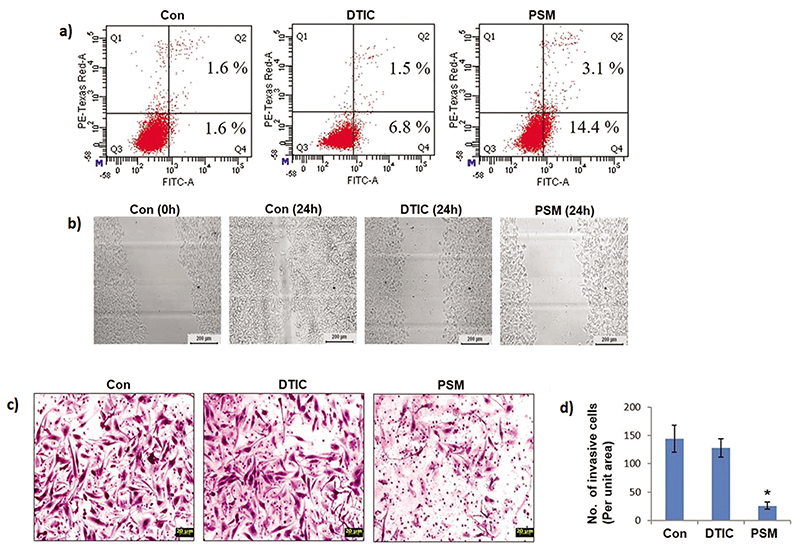
a) Apoptosis results of control, DTIC- and PSM-treated B16F10 cells; b) results of the scratch assay; c) representative images of the Boyden chamber assay; d) quantitative analysis of the Boyden chamber assay (*p < 0.05). Reproduced with permission.^[264]^ Copyright 2019, The Royal Society of Chemistry.

The same group also comparatively studied PSM with respect to an approved chemotherapeutic for metastatic melanoma (dacarbazine, DTIC) to treat melanoma and to reduce nodule formation in lung metastasis through immunomodulatory properties.^[264]^ In vitro studies in B16F10 mouse melanoma cells showed anti-invasive and strong apoptotic effects compared to DTIC (Figure 7). A reduction in tumor volume and in the number of lung nodules was observed in tumor-bearing mice. In addition, the enhancement of IL-2, TNF-α, and IFN-γ confirmed the immunotherapeutic potential of PSM in vivo. Antigen-specific IFN-γ-producing T cells and tumor-infiltrating CD8^+^ T cells increased after PSM treatment, which is an indicator of improved overall survival and metastasis inhibition.^[264]^

**Figure 8:**
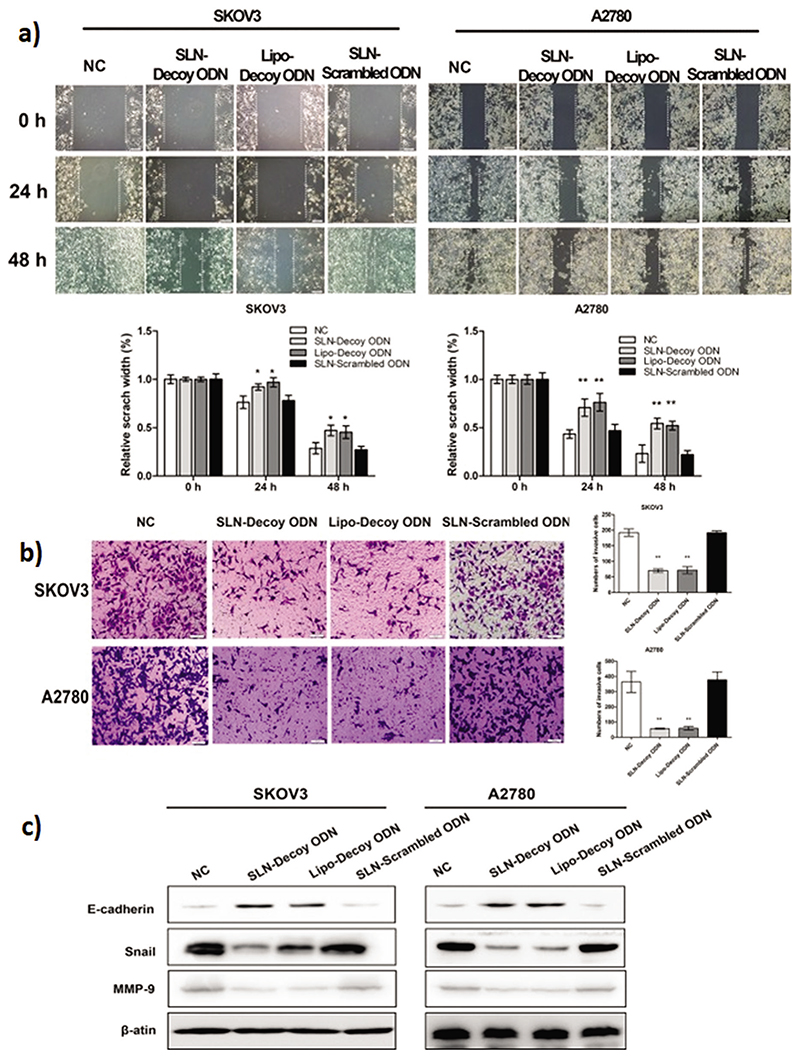
a) Scratch assay, b) transwell invasion assay, c) Western blot analysis of E-cadherin, Snail, and MMP-9 (*p < 0.05, **p < 0.01) Reproduced under the terms of the CCA 4.0 International Licence.^[265]^ Copyright 2015, Public Library of Science.

In another study, SLNs were prepared using signal transducer and activator of transcription 3 (STAT3) decoy ODN, which can suppress tumor growth, and their in vitro behavior was studied on human ovarian cancer cell lines (A2780 and SKOV3).^[265]^ Since plain decoy ODN is rapidly degraded in cells, SLNs are used for safe and efficient gene delivery. The upregulation of Bax, cleaved caspase 3, beclin-1, and LC3-II expression, and the downregulation of Bcl-2, pro-caspase 3, Survivin, p-Akt, and p-mTOR expression are associated with apoptotic and autophagic cell death. Furthermore, reduced migration and invasion, increased E-cadherin expression, and decreased Snail and MMP-9 expression were observed in SLN-STAT3 decoy ODN-treated cells (Figure 8).^[265]^

#### 4.3.3 NLCs

NLCs are second-generation lipid carriers that consist of a mixture of solid and liquid lipids in various ratios. They were developed to overcome the limitations of SLNs, such as low drug loading capacity and drug escape from the lipid matrix during storage.^[266]^

Nordin et al. showed the antimetastatic and antitumor effects of citral-loaded NLCs (Citral-NLCs) in vitro on MDA MB-231 cells.^[267]^ Citral reduces breast cancer growth by decreasing aldehyde dehydrogenase 1A3 (ALDH1A3)-mediated colony formation.^[268]^ The antimetastatic activity of Citral-NLCs was tested using in vitro invasion, scratch, and migration assays, and the results showed a significant decrease in migrated and invaded cells due to the upregulation of Bax, cleaved caspase 3, TRAIL R1, and cytochrome C combined with the downregulation of the procaspase 3, Bcl-2, Bcl-X, and survivin. Overall, the data indicated that Citral-NLCs show antimetastatic and antiangiogenic activity by regulating various signaling pathways related to metastasis, apoptosis, and cell cycle.^[267]^

The same group further studied the antimetastatic and antiangiogenic activity of Citral-NLCs in 4T1 tumor-bearing BALB/c mice in vivo by oral administration.^[269]^ It was found that Citral-NLCs inhibits metastasis in lung and bone marrow through downregulation of metastasis-related gene expression, such as MMP-9, ICAM, inducible nitric oxide synthase (iNOS), and nuclear factor kappa B (NF-kB), and of angiogenesis-related proteins, such as granulocyte colony-stimulating factor (G-CSF) alpha, eotaxin, bFGF, VEGF, IL-1alpha, and macrophage colony-stimulating factor (M-CSF). Additionally, the level of metastasis-related cytokines was evaluated, and the results showed decreased IL-1*β* and IL-6 secretion. This study suggests the use of Citral-NLCs against triple-negative breast cancer, owing to the inhibition of the proliferation and invasiveness of 4T1 cells in vitro, and to apoptosis-related inflammation and metastasis inhibition in a 4T1-induced breast cancer mouse model.^[269]^

In another study, thymoquinone (TQ) was loaded into NLCs (TQ-NLCs) to observe the anticancer activity with respect to the commercial drug DOX in 4T1 tumor-bearing BALB/c mice.^[270]^ The prepared lipid carrier system and TQ were orally administered to the mice, and the survival rate increased when NLCs were used. The inhibition of lung metastasis was observed even at low concentrations of TQ-NLCs, and TQ-treated mice were compared to DOX-treated mice in terms of downregulation of MMP-2 expression and upregulation of apoptotic pathways such as Bcl-2, Bax and caspase 8.^[270]^

A comparative study of DOX-loaded NLCs (DOX–NLCs) and DOX-loaded liposomes (DOX-liposomes) was carried out to analyze the antitumor and antimetastatic effects of DOX in a breast cancer animal model (4T1 tumor model).^[271]^ The prepared systems were i.v. administered, and the results compared to those obtained with free DOX. DOX–NLCs showed the best outcome in terms of tumor growth with respect to DOX-liposomes and free DOX. In addition, lung metastasis was prevented in both DOX–NLCs and DOX-liposomes treatments, assuming that they prevent the dissemination of cells from the primary tumor rather than exerting a direct cytotoxic effect on micrometastases.^[271]^

**Figure 9:**
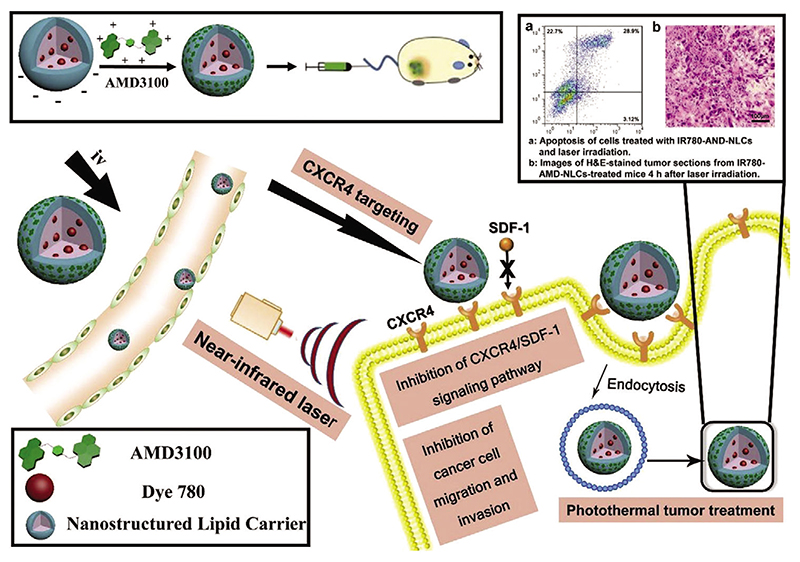
Schematic representation of IR780-AMD-NLCs. Reproduced underthe terms ofthe CCA4.0 International Licence.^[272]^ Copyright 2017, Elsevier.

The combined effect of IR780 (NIR dye)-loaded and AMD3100 (a small molecule chemokine receptor CXCR4 antagonist, which is important for the prevention of cancer metastasis)-coated NLCs was studied with the aim to decrease cancer invasiveness and to enhance tumor targeting and photothermal therapeutic outcomes in metastatic breast cancer (Figure 9).^[272]^ The prepared lipid-based system (IR780-AMD-NLCs) inhibited cancer cell invasion and lung metastasis in vitro *(4T1-luc* cells) and in vivo *(4T1-luc* tumor-bearing BALB/c mice). The photothermal efficiency was enhanced by the incorporation of IR780 into NLCs with respect to free IR780; furthermore, in vivo biodistribution and imaging studies showed that AMD3100-coated NLCs accumulated at high levels in the tumor site. Free IR780/laser and IR780-NLCs/laser groups presented inhibition of lung metastasis due to PTT, which killed or irreversibly damaged the cancer cells suggesting AMD3100-coated NLCs for both metastasis prevention and tumor therapy.^[272]^

Table 2, as a conclusion, summarizes nanomaterial-based strategies exploited against cancer metastasis and neoangiogenesis.

## 5 Conclusions and Future Perspectives

Metastasis treatment is a great challenge in scientific and clinical research because it is a major cause of morbidity and mortality in cancer patients. There is an increase in the cancer survival rate due to earlier diagnosis and inhibition of tumor progression; however, limited treatments have been elaborated for cancer metastasis and angiogenesis, in particular because the underlying mechanisms remain poorly understood. Current therapies used to treat cancer metastasis are represented by conventional approaches (surgical removal, chemotherapy, and radiotherapy), and still present several problems that result in negative outcomes.

Although many nanotherapeutics have focused on primary cancer treatments, as summarized in this review they also have the potential to combat the metastatic spread of malignant tumors. Despite the advancements in this field, side effects on healthy and primary cancer cells still need to be deeply investigated. For example, inorganic nanomaterials are commonly used in cancer therapy, particularly for hyperthermia, photodynamic therapy, and drug delivery; however, one of the bottlenecks of inorganic nanomaterials is their accumulation in reticuloendothelial system, resulting into long-term toxic effects. The other significant issue to be addressed is related to the targeting of the therapy to metastatic sites. Thanks to the tailored and tunable theranostic functions of nanomaterials, therapeutics can be targeted, their release can be controlled, and the prolonged half-life of the drug can improve the success of the treatment. However, additional studies are required to develop robust methods to precisely target the metastatic tumor microenvironment.

In addition, the advances in cancer immunotherapy show promising results in patients suffering from metastasis, yet it should be noted that the tumor type, stage, and location strongly affect the immune response. Lack of understanding of the relation between metastasis and immune response in preclinical studies indicates the need of further investigations in this direction.

Although most of the studies summarized in this review showed promising results, they are still in preclinical phase, and only a few nanoformulations have been approved and placed in the clinics for specific cancer treatments. Limited cancer models mimicking actual pathological situation still represent a challenge that needs to be addressed for the successful applications of cancer nanomedicine in clinical oncology. We firmly believe that an improvement in cancer biology knowledge, jointly to a multidisciplinary approach including clinicians and nanotechnology scientists, will shape the future treatment strategies in cancer treatments.

From a scientific perspective, if we succeed in gaining a better understanding of the biological and molecular mechanisms at the base of metastasis and angiogenesis, we could identify high potentially beneficial cellular and molecular targets. Understanding the mechanisms regulating the modulation of metastasis and of apoptosis pathways will be critical in designing effective strategies for the development of novel targeted molecular therapeutics. On the side of novel therapeutics, it is clear that single-step antiangiogenic or antimetastatic approaches cannot be sufficient as standing-alone strategies, and new-generation combinational nanomaterial-based platforms should be elaborated to face distinct aspects of metastasis and angiogenesis.

## Figures and Tables

**Table 1:** Common methods used for assessing cancer metastasis and angiogenesis.

Assays	Advantages	Disadvantages
Scratch/wound healing assay	Low cost Easy implementation	Suitable just for adherent cell lines Not suitable for assessment of chemotaxis Needs personnel practice Injured cells in border cause unreliable migration profile Nonreversible biochemical conditions
Gap closure assay	Low cost Easy implementation Provides healthy cells in wound border	Suitable just for adherent cell lines Nonreversible biochemical conditions Not suitable for assessment of chemotaxis
Phagokinetic track assay	Low cost Easy implementation Detectable migration speed and angle	Suitable just for adherent cell lines Not available for assessment of chemotaxis Not suitable for large-scale investigations Colloidal gold exposure of cells can cause misleading of results
Transwell/modified Boyden chamber Migration Invasion Transendothelial migration	Sensitivity to low levels of chemoattractants Available for adherent and nonadherent cell lines	High cost of membrane Assessed for an only fraction of total membrane surface Changing optimal time depends on cell type Difficulties in maintaining transfilter gradient prolonged time
Microfluidic-based assays	Flexibility of controlling environmental conditions Static flow conditions mimic circulatory system Maintaining transfilter gradient in static fluid flow	
Multiphoton imaging	Generation of second harmonic Minimal photobleaching and toxicity	High cost Technology still in development Not used clinically
OFDI	Exogenous contrast reagents not required Lack of photobleaching or toxicity Enhanced depth penetration	High cost Technology still in development Not used clinically
Whole body fluorescent imaging	Noninvasive	Not used clinically
MR	Used clinically	High cost High contrast only generated in soft tissues Long imaging times required for high resolution
CT	Used clinically	High cost High contrast generated in lungs and bones High radiation doses needed for high resolution.
PET	Used clinically	High cost Limited detection capacity
Endothelial cell proliferation assay		
Cell counting	Easy implementation Low cost	Limited information about proliferation and apoptosis
DNA synthesis detection	Radioactive labeling not required	Time consuming and difficult quantification
Colorimetric cell counting	Low cost Indicator of membrane integrity	Limited information about proliferation and apoptosis High possible cell counting errors
Matrix degradation assay	Low cost Information about relative identity of MMPs	Time consuming Not adaptable to large-scale investigations
Endothelial cell migration assay	Low cost Discrimination chemotaxis or directed migration Examining recruitment of mural cells	Low sensitivity Difficult quantification Not suitable for large scale investigations
Endothelial cell differentiation assay	Detailed monitoring of endothelial cell differentiation	Weak mimicking of systemic parameters
Matrigel plug assay	Easy implementation Low interference to natural environment of cells	Limited by the number of animals
Sponge/matrigel assay	High precise visualization of angiogenic response	Time consuming Limited by the number of animals
Alginate microbead release assay	Protects cells from host organism immune attack Slow degradation Slow drug release	Difficulties in endothelial cell-including alginate microbead preparation Limited by the number of animals
Hollow fiber assay	More suitable than surface assays for tumor angiogenesis Permits long-term observation Well-tolerated procedure	Nonspecific inflammatory response Not a typical site for pathological angiogenesis in vivo Not suitable for noninvasive monitoring

**Table 2:** Nanomaterials used in cancer metastasis and neoangiogenesis treatment.

Nanomaterial	Anticancer agent	Targeting agent	Investigated disease model	Treatment mechanisms	Ref.
AuNPs	—	—	A2780, OVCAR5, SKOV3-ip and OSE cell lines, and mouse model of ovarian cancer	Antiangiogenic activity by upregulation of E-cadherin and serpin E1, downregulation of N-cadherin, vimentin and Snail	[161]
AuNPs	—	PEG, RGD and NLS peptides	HEYA8 cells	Decreased cell migration by increasing the expression of lamin A/C protein level	[162]
AuNPs	—	—	PC3 and HDF cells	Decreased cell migration	[163]
AuNRs	—	RGD peptide	HSC-3 cells	Decreased cell migration after PTT by downregulation ofintegrins, and actin, microtubule, Rho GTPases, and kinases-related signaling pathways	[164]
mPEG-GNRs@BSA/R837	R837	*m*PEG	B16-F10 cells and C57/BL6 mice	Inhibition of lung metastasis as a result of immune responses through increasing levels ofTNF-α, IL-6, and IL-12	[166]
AuNRs	—	—	PC3, MDA-MB-231, and B16F10 cells	Inhibiting in vitro cell migration and invasion by downregulating the expression of *OXPHOS* and glycolysis	[167]
CoFe_2_O_4_@MnFe_2_O_4_ nanoparticles	—	—	4T1, HUVECs, and U87 cells, BALB/c nude mice	Combined magnetic hyperthermia and immune therapy and activation of dendritic and cytotoxic T cells	[172]
MNPs	—	—	4T1, CT26, and SC-1 cells, BALB/c mice	Increased MHT killing capacity due to the activation of antioxidant proteins, low expression of HSPs, and increased cell death with temperature	[173]
PEI-SPIONs	—	PEI	SVEC4-10, MDA-MB-231, THP-1, and HUVECs, MDA-MB-231 xenograft-bearing nude mice	Impaired angiogenesis altering actin polymerization and proangiogenic cytokines, and in vivo reduced blood vessels and promotion of macrophage infiltration	[174]
Octagonal-shaped iron oxide nanoparticles	—	—	Panc02 and NCTC1469 cells, and leptin-deficient transgenic obese mice	Noninvasively detection ofTAMs with high sensitivity and selectivity	[175]
QDs@Gd3^+^-NGR	—	Asn–Gly–Arg (NGR)	PANC-1 cells	Inhibiting cell proliferation, invasion and metastasis due to increased ROS and apoptosis	[178]
Tan-ZnO QDs	Tangeretin (Tan)	—	H358 cells	Decreased migration due to the regulation of apoptotic Bax and Bcl-2 proteins, G2/M phase arrest, downregulation of MMP2, MMP9, and VEGF; improved tracking during chemotherapy	[179]
Ag_2_S QDs	—	PEG	Nude mice	Real-time imaging of tumor angiogenesis due to deep tissue penetration, high spatial and temporal resolution, and low autofluorescence	[180]
CdSe/ZnS or InP/ZnS QDs	DC vaccines	PDMAEMA-*b*-PEG	B16–F10 tumor bearing C57BL/6j mice	Inhibition of lung metastasis due to polarization of TAMs caused by loss of CCL3 chemokine ligand	[182]
CdSe/ZnS QDs	—	PEG	MSCs, MDA-MB-231, and MCF-10A cells; SCID mice	Selectively targeting and imaging of tumor and metastatic tissue	[183]
Hollow MSNs	DOX, ATRA, and IL-2	Lipid coated	L929 cellsand B16F10 tumor-bearing mice	Decreased metastasis by activating T lymphocytes and NK cells, promoting IFN-*y* and IL-12 cytokines secretion, downregulating cytokine IL-10 and TGF-*β*, regulating myeloid-derived suppressor cells-induced immunosuppression	[186]
Silica nanoparticles	—	Cy3(+) and Cy5(+)	MDA-MB-231 cells and athymic nude-Foxn1^nu^ mice	Simultaneous imaging of cells, bone marrow, and mineralized matrix in bone metastasis due to electron-dense structure of silica nanoparticles	[188]
MSNs	—	—	HMMEC	Antiangiogenic activity by generation of ROS, which interfere with p53 tumor suppressor pathway	[187]
MWNTs, C_60_ fullerenes, and graphite	—	—	HUVECs	Antiangiogenic efficiency by inhibiting HUVEC proliferation	[192]
Graphene nanosheets, MWNTs, C_60_ fullerenes, diamond, and graphite nanoparticles	—	—	In ovo chick embryo chorioallantoic membrane model	VEGF secretion-dependent antiangiogenic efficiency by diamond nanoparticle and MWNT; fullerene-dependent blood vessel stimulation	[193]
Chitosan	—	—	HCC and BEL-7402 cells xenograft-bearing nude mice	VEGFR2 production correlated with antiangiogenic activity	[210]
Chitosan	siRNA (survivin gene)	PEG	4T1 cells	Inhibition of tumor growth by silencing survivin gene	[212]
DOC-heparin			Athymic BALB/c-nu/nu female nude mice xenograft models	Angiogenic inhibition of angiogenesis following angiogenic factors binding	[217]
Cholesterol-conjugated LMWH (LHC)	DOX		4T1 cells and 4T1-luc cells, and female BALB/c mice	Growth factor receptor affinity-related angiogenic inhibition	[220]
N-acetyl glucosamine sulfonated hyaluronic acid	—	—	HUVECs	Strong affinity to VEGF165a resulted in decrement of HUVEC survival of HMVEC tube formation	[223]
Cross-linked hyaluronic acid gel (CHAG)	—	—	AGS, HepG2 cell lines and SGC-7901 xenograft nude mice	Downregulation ofintegrin, EGFR, and VEGFR-2	[224]
HPMA	TNP-470	ALN	K7M2 murine osteosarcoma cells, xenograft of mice bearing mCherry-labeled K7M2 murine osteosarcoma	Inhibited tumor-induced neovascularization by osteosarcoma targeted cytotoxic agents	[233]
HPMA	TNP-470	ALN	Immunodeficient (SCID) male mice inoculated with mCherry-labeled MG-63-Ras human osteosarcoma	VEGF-induced vascular hyperpermeability reduction and osteosarcoma inhibition	[234]
HPMA	Paclitaxel	ALN	HUVECs	VEGF and tubular structure inhibition, providing migration reduction	[235]
PMLA-PEI-TAT@PEG-DMMA	DOX	—	A549 and MHCC97-H cells and A549 xenograft mouse	Tumor growth inhibition induced by cellular toxicity	[238]
PGA	Paclitaxel	Cyclic RGD peptidomimetic ((RGDfK)2)	U87-MG, 4T1, and HUVECs	Inhibition of endothelial cell migration towards VEGF and attachment to fibrinogen, blocked capillary-like tube formation	[240]
PGA/PEG	—	—	Cocultured A549 and Jurkat E6.1 lymphocytes	Selective accumulation in a 3D model of metastatic lymph nodes	[241]
PLGA	Temozolomide, vatalanib	—	C6 glioma cells	Tumor growth inhibition mediated by apoptosis mechanisms	[244]
Liposomes	DOX	PC-SA	B16F10 cells and C57BL/6 mice	Activating the immune system against lung metastasis by the upregulation of ConA-specific Th1 cytokines	[253]
Liposomes	Paclitaxel	Glutamic oligopeptides-RGD peptide	MDA-MB-231 cells and Balb/c nu mice	Superior targeting ability and favored accumulation of paclitaxel in bone metastasis due to the synergistic effect of the dual-mediated endocytosis	[254]
Liposomes	DOX	ICAM1 and EGFR	MDA-MB-231, MDA-MB-436, MDA-MB-157, MCF10A, and MDA-MB-231 tumor-bearing mice	Inhibition of orthotopic and lung metastasis by blockage of EGFR and ICAM1 signaling cascades simultaneously	[255]
Liposomes	DOX and IND	—	4T1 orthotopic tumor bearing mice	Immune response mediated eradication of lung metastasis through PD-1 blocking antibodies	[257]
Liposomes	PEDF and DNA	iRGD	CT26 and HUVECs, and BALB/c mice	Strong inhibition of metastatic tumor nodules in lung due to decreased migration and invasion and to apoptosis	[258]
Liposomes	MATT	—	4T1 and MDA-MB-435 cells, and 4T1 tumor-bearing mice	Antimetastatic and antiangiogenic by downregulation of *MMP-2* and *MMP-9* expression	[259]
SLNs	Docetaxel	—	4T1, MCF7, and NIH-3T3 cells, and 4T1-bearing BALB/c mice	Prevention of lung metastasis by inducing apoptosis and microtubule damage and by decreasing IL-6 production, and BCL-2 and Ki-67 expression	[262]
SLNs	Paclitaxel	TOC and PEG	C6 glioma and NIH 3T3 cells, and orthotopic glioma-bearing S/D rats	Enhanced antiangiogenic activity due to decreased CD31 expression	[263]
SLNs	Paclitaxel	TOC and PEG	B16F10 cells and C57BL/6 mice	Reduction in number of metastatic lung nodules following increment of antigen-specific IFN-*y* producing T cells and of tumor-infiltrating CD8^+^ T cells	[264]
SLNs	STAT3 decoy ODN	—	A2780and SKOV3 cells	Reduced migration and invasion due to increased E-cadherin expression and decreased Snail and MMP-9 expression	[265]
NLCs	Citral	—	MDA MB-231 cells	Antimetastatic and antiangiogenic activity by upregulation of Bax, cleaved caspase 3, TRAIL R1 and cytochrome C, and downregulation of pro-caspase 3, Bcl-2, Bcl-X, and survivin	[267]
NLCs	Citral	—	MDA MB-231 and MCF-10A cells; 4T1 tumor-bearing BALB/c mice	Inhibition of metastasis in lung and bone marrow through downregulation of MMP-9, ICAM, iNOS, NF-k*β*, G-CSF alpha, eotaxin, bFGF, VEGF, IL-1α, and M-CSF	[269]
NLCs	Thymoquinone	—	4T1 tumor-bearing BALB/c mice	Inhibition of lung metastasis due to downregulation of MMP-2 expression and upregulation of Bcl-2, Bax, and caspase 8 pathways	[270]
NLCs and liposomes	DOX	—	4T1 tumor-bearing BALB/c mice	Prevention of metastasis by avoiding the dissemination of cells from the primary tumor	[271]
NLCs	IR780	AMD3100	4T1-luc cells, and 4T1-luc tumor-bearing BALB/c mice	Inhibition of invasion and lung metastasis by photothermal effects	[272]
